# Integration of p16/HPV DNA Status with a 24-miRNA-Defined Molecular Phenotype Improves Clinically Relevant Stratification of Head and Neck Cancer Patients

**DOI:** 10.3390/cancers14153745

**Published:** 2022-07-31

**Authors:** Julia Hess, Kristian Unger, Cornelius Maihoefer, Lars Schüttrumpf, Peter Weber, Sebastian Marschner, Ludmila Wintergerst, Ulrike Pflugradt, Philipp Baumeister, Axel Walch, Christine Woischke, Thomas Kirchner, Martin Werner, Kristin Sörensen, Michael Baumann, Ingeborg Tinhofer, Stephanie E. Combs, Jürgen Debus, Henning Schäfer, Mechthild Krause, Annett Linge, Jens von der Grün, Martin Stuschke, Daniel Zips, Martin Canis, Kirsten Lauber, Ute Ganswindt, Michael Henke, Horst Zitzelsberger, Claus Belka

**Affiliations:** 1 Research Unit Radiation Cytogenetics, Helmholtz Zentrum München, German Research Center for Environmental Health GmbH, 85764 Neuherberg, Germany; unger@helmholtz-muenchen.de (K.U.); peter.weber@helmholtz-muenchen.de (P.W.); schneider_ludmila@yahoo.de (L.W.); zitzelsberger@helmholtz-muenchen.de (H.Z.); 2 Clinical Cooperation Group “Personalized Radiotherapy in Head and Neck Cancer”, Helmholtz Zentrum München, German Research Center for Environmental Health GmbH, 85764 Neuherberg, Germany; cornelius.maihoefer@gmail.com (C.M.); lschuettrumpf@gmail.com (L.S.); sebastian.marschner@med.uni-muenchen.de (S.M.); ulrike.pflugradt@med.uni-muenchen.de (U.P.); philipp.baumeister@med.uni-muenchen.de (P.B.); martin.canis@med.uni-muenchen.de (M.C.); kirsten.lauber@med.uni-muenchen.de (K.L.); ute.ganswindt@i-med.ac.at (U.G.); claus.belka@med.uni-muenchen.de (C.B.); 3 Department of Radiation Oncology, University Hospital, LMU Munich, 81377 Munich, Germany; 4 Department of Otorhinolaryngology, Head and Neck Surgery, University Hospital, Ludwig-Maximilians-University of Munich, 81377 Munich, Germany; 5 Research Unit Analytical Pathology, Helmholtz Zentrum München, German Research Center for Environmental Health GmbH, 85764 Neuherberg, Germany; axel.walch@helmholtz-muenchen.de; 6 Institute of Pathology, Faculty of Medicine, Ludwig-Maximilians-University of Munich, 81377 Munich, Germany; christine.woischke@med.uni-muenchen.de (C.W.); thomas.kirchner@med.uni-muenchen.de (T.K.); 7 German Cancer Consortium (DKTK), Partner Site Munich, and German Cancer Research Center (DKFZ), 69120 Heidelberg, Germany; stephanie.combs@tum.de; 8 Institute for Surgical Pathology, Medical Center-University of Freiburg, 79106 Freiburg, Germany; martin.werner@uniklinik-freiburg.de (M.W.); kristin.werner@uniklinik-freiburg.de (K.S.); 9 Faculty of Medicine, University of Freiburg, 79106 Freiburg, Germany; 10 German Cancer Consortium (DKTK), Partner Site Freiburg, and German Cancer Research Center (DKFZ), 69120 Heidelberg, Germany; henning.schaefer@uniklinik-freiburg.de (H.S.); michael.henke@uniklinik-freiburg.de (M.H.); 11 German Cancer Consortium (DKTK), Partner Site Dresden, and German Cancer Research Center (DKFZ), 69120 Heidelberg, Germany; michael.baumann@dkfz-heidelberg.de (M.B.); mechthild.krause@uniklinikum-dresden.de (M.K.); annett.linge@uniklinikum-dresden.de (A.L.); 12 German Cancer Research Center (DKFZ), 69120 Heidelberg, Germany; 13 OncoRay—National Center for Radiation Research in Oncology, Faculty of Medicine and University Hospital Carl Gustav Carus, Technische Universität Dresden, 01309 Dresden, Germany; 14 Department of Radiooncology and Radiotherapy, Charité University Hospital Berlin, 10117 Berlin, Germany; ingeborg.tinhofer@charite.de; 15 German Cancer Consortium (DKTK), Partner Site Berlin, and German Cancer Research Center (DKFZ), 69120 Heidelberg, Germany; 16 Department of Radiation Oncology, Klinikum rechts der Isar, Technische Universität München, 81675 Munich, Germany; 17 Institute of Radiation Medicine (IRM), Helmholtz Zentrum München, German Research Center for Environmental Health GmbH, 85764 Neuherberg, Germany; 18 Department of Radiation Oncology, Heidelberg Ion Therapy Center (HIT), University of Heidelberg, 69120 Heidelberg, Germany; juergen.debus@med.uni-heidelberg.de; 19 German Cancer Consortium (DKTK), Partner Site Heidelberg, and Clinical cooperation unit Radiation Oncology, German Cancer Research Center (DKFZ), 69120 Heidelberg, Germany; 20 Department of Radiation Oncology, Medical Center, Faculty of Medicine, University of Freiburg, 79106 Freiburg, Germany; 21 Department of Radiotherapy and Radiation Oncology, Faculty of Medicine and University Hospital Carl Gustav Carus, Technische Universität Dresden, 01307 Dresden, Germany; 22 National Center for Tumor Diseases (NCT), Partner Site Dresden, 01307 Dresden, Germany; 23 Helmholtz-Zentrum Dresden—Rossendorf, Institute of Radiooncology—OncoRay Dresden, 01328 Dresden, Germany; 24 Department of Radiotherapy and Oncology, Goethe University Frankfurt, 60596 Frankfurt, Germany; jens.vondergruen@kgu.de; 25 German Cancer Consortium (DKTK), Partner Site Frankfurt, and German Cancer Research Center (DKFZ), 69120 Heidelberg, Germany; 26 Department of Radiotherapy, Medical Faculty, University of Duisburg-Essen, 45147 Essen, Germany; martin.stuschke@uk-essen.de; 27 German Cancer Consortium (DKTK), Partner Site Essen, and German Cancer Research Center (DKFZ), 69120 Heidelberg, Germany; 28 Department of Radiation Oncology, Faculty of Medicine and University Hospital Tübingen, Eberhard Karls University Tübingen, 72076 Tübingen, Germany; daniel.zips@med.uni-tuebingen.de; 29 German Cancer Consortium (DKTK), Partner Site Tübingen, and German Cancer Research Center (DKFZ), 69120 Heidelberg, Germany; 30 Department of Therapeutic Radiology and Oncology, Innsbruck Medical University, 6020 Innsbruck, Austria

**Keywords:** head and neck cancer, HNSCC, HPV, miRNA, signature, prediction, prognosis, mRNA, interaction, signaling pathways

## Abstract

**Simple Summary:**

Human papillomavirus (HPV)-driven head and neck squamous cell carcinomas (HNSCC), regarded as a distinct clinical entity, are characterized by a considerably favourable prognosis after radio(chemo)therapy and a not yet fully understood distinct molecular pathogenesis. We aimed to develop a miRNA-signature that identifies HPV-associated HNSCC according to their specific molecular pathogenesis, and to characterise the transcriptome compared to HPV-negative HNSCC. We performed miRNA expression profiling of *n* = 229 HPV characterized HNSCC specimens of patients treated by adjuvant radio(chemo) therapy. Using lasso-regression, a 24-miRNA signature predicting HPV-status was built in a multicentre cohort and validated in a single-centre cohort. Its combination with p16/HPV DNA status improved clinically relevant risk stratification, allowed the identification of an HPV-associated patient subgroup with impaired overall survival, and might be considered for future clinical decision-making. miRNA-transcriptome integration identified HPV-specific signaling pathways.

**Abstract:**

Human papillomavirus (HPV)-driven head and neck squamous cell carcinomas (HNSCC) generally have a more favourable prognosis. We hypothesized that HPV-associated HNSCC may be identified by an miRNA-signature according to their specific molecular pathogenesis, and be characterized by a unique transcriptome compared to HPV-negative HNSCC. We performed miRNA expression profiling of two p16/HPV DNA characterized HNSCC cohorts of patients treated by adjuvant radio(chemo)therapy (multicentre DKTK-ROG *n* = 128, single-centre LMU-KKG *n* = 101). A linear model predicting HPV status built in DKTK-ROG using lasso-regression was tested in LMU-KKG. LMU-KKG tumours (*n* = 30) were transcriptome profiled for differential gene expression and miRNA-integration. A 24-miRNA signature predicted HPV-status with 94.53% accuracy (AUC: 0.99) in DKTK-ROG, and 86.14% (AUC: 0.86) in LMU-KKG. The prognostic values of 24-miRNA- and p16/HPV DNA status were comparable. Combining p16/HPV DNA and 24-miRNA status allowed patient sub-stratification and identification of an HPV-associated patient subgroup with impaired overall survival. HPV-positive tumours showed downregulated *MAPK*, *Estrogen*, *EGFR*, *TGFbeta*, *WNT* signaling activity. miRNA-mRNA integration revealed HPV-specific signaling pathway regulation, including *PD−L1 expression/PD−1 checkpoint pathway in cancer* in HPV-associated HNSCC. Integration of clinically established p16/HPV DNA with 24-miRNA signature status improved clinically relevant risk stratification, which might be considered for future clinical decision-making with respect to treatment de-escalation in HPV-associated HNSCC.

## 1. Introduction

High-risk human papillomavirus (HPV) driven head and neck squamous cell carcinoma (HNSCC), regarded as a distinct clinical entity, is characterized by a considerably favourable prognosis after radio(chemo)therapy and a not yet fully understood distinct molecular pathogenesis [1,2,3,4]. The complex and heterogenous mutation and aberration patterns in HPV-related and HPV-negative HNSCC—mainly caused by tobacco and alcohol consumption—affect all molecular levels, including microRNAs (miRNA). MiRNAs act as important post-transcriptional regulators, and thereby interfere with multiple signaling pathways. Deregulation and HPV association of miRNAs has been shown in several HNSCC studies [1,5,6,7,8,9,10,11,12,13]; however, overlaps between studies and independent validation in well-characterized HNSCC sets are sparse.

Here, applying comprehensive miRNA profiling, we characterized HPV-positive and HPV-negative HNSCC specimens (*n* = 229) of a multicentre and a single centre cohort, both comprising patients treated by adjuvant radio(chemo)therapy. We aimed to develop a miRNA signature that identifies HPV-positive HNSCCs according to their specific molecular pathogenesis, and to characterise the transcriptome compared to HPV-negative HNSCCs.

## 2. Materials and Methods

### 2.1. Patient Cohorts

Our study was conducted in compliance with the REMARK guidelines and fulfils the requirements defined by Simon et al. 2009 [14,15]. Two independent HNSCC cohorts were included: the multicentre DKTK-ROG (German Cancer Consortium Radiation Oncology Group; *n* = 128; [16]) and the single-centre LMU-KKG cohort (Ludwig-Maximilians-University of Munich, Clinical Cooperation Group “Personalized Radiotherapy in Head and Neck Cancer”; *n* = 101; [17,18]). All patients were diagnosed with histologically proven HNSCC of the hypopharynx, oropharynx, or oral cavity, and received adjuvant radio(chemo)therapy as a curative approach after surgical resection. This retrospective study was conducted in accordance with the Declaration of Helsinki. Ethical approval (EA) was obtained by the ethics committees of all DKTK-ROG partners, including the LMU (EA 312-12, 448-13, 17-116).

The DKTK-ROG cohort originally comprised 221 HNSCC patients who were treated at one of eight different DKTK partner locations [16]. Inclusion criteria were positive microscopic resection margins and/or extracapsular extension (ECE) of lymph nodes, and/or tumour stage pT4, and/or more than three positive lymph nodes. This study reports on a subgroup of 128 patients with available tumour material treated between 2004 and 2011. All DKTK-ROG patients received postoperative radiotherapy covering the previous tumour region, and regional lymph nodes with concomitant cisplatin (CDDP)-based chemotherapy according to standard protocols. Adjuvant radiotherapy, including elective irradiation of cervical lymph nodes, was applied with a median dose of 50 Gy (median dose 2 Gy/fraction), and a boost to the former tumour region, and to microscopic disease (if any) to a median dose of 66 Gy (median dose 2 Gy/fraction).

The LMU-KKG cohort included all HNSCC patients treated with adjuvant radio(chemo)therapy at the Department of Radiation Oncology, LMU, Germany, between 06/2008 and 01/2013 with available tumour tissue specimens [17]. A median radiation dose of 64 Gy (2 Gy/fraction, five fractions/week.) was applied to the former tumour bed or regions with extracapsular extension (ECE). Elective lymph node regions were irradiated with a median dose of 50 Gy (2 Gy/fraction) depending on tumour stage and localization; 56 Gy (2 Gy/fraction) were applied to the affected lymph node regions. In case of close (R0, <5 mm) or positive microscopic resection margins and/or ECE, patients (64.4%) received simultaneous chemotherapy. Of the patients, 77% received CDDP/5-fluorouracil (5-FU). In a few cases, Mitomycin C (MMC), 5-FU/MMC or Cetuximab was used instead of platinum-based chemotherapy.

Tumour stage was assessed using the UICC TNM *Classification of Malignant Tumours*, 7th edition.

Median time from diagnosis to the first day of radiotherapy treatment was 66 days (IQR 52–80) for the DKTK-ROG and 68 days (IQR 55–83) for the LMU-KKG cohort. Median time from surgical tumour resection to the first day of radiotherapy treatment was 46 days (IQR 37–54) for the DKTK-ROG and 42 days (IQR 34–51) for the LMU-KKG cohort.

### 2.2. Tumour Specimens

Formalin-fixed paraffin-embedded tumour specimens were derived from treatment-naive surgically resected tumour tissues. Haematoxylin-eosin-stained tissue sections were histopathologically reviewed by a pathologist (DKTK-ROG: KS; LMU-KKG: CW/AW). The tumour area was defined and micro-dissected, whereby only samples with at least 60% tumour cells were included. Total RNA including the small RNA fraction was extracted using the Qiagen miRNeasy FFPE (DKTK-ROG) or the AllPrep DNA/RNA FFPE Kit (LMU-KKG) according to the manufacturer’s protocols (Qiagen, Hilden, Germany).

### 2.3. HPV Characterisation—LMU-KKG Cohort

#### 2.3.1. p16/HPV DNA Status

p16/HPV DNA status of LMU-KKG specimens was determined by p16^INK4a^ immunohistochemistry (IHC) in combination with HPV DNA detection as described previously [19]. p16^INK4a^ staining was performed using the CINtec^TM^ Histology Kit (Roche mtm laboratories AG, Mannheim, Germany) on a Ventana Benchmark LT automated immunostainer (Ventana Medical Systems, Tucson, AZ, USA) according to the manufacturer’s protocol. As positive and negative controls, FFPE sections from embedded p16-positive (UPCI SCC154) and p16-negative HNSCC cell lines (Cal33) were used. Tumour specimens with strong and diffuse nuclear and cytoplasmic staining in more than 70% of tumour cells were classified as p16-positive, while tissue with only weak diffuse or absent staining was considered p16-negative [20]. Evaluation of p16^INK4a^-stained tissue sections was performed by two independent observers. Detection of mucosotropic HPV DNA was performed by quantitative real-time PCR (q-PCR) in combination with SYBR green chemistry (Clontech Laboratories, Inc., Mountain View, CA, USA. Genomic DNA (50 ng)was subjected to q-PCR reactions (10 µL) on a ViiA 7 q-PCR system (Thermo Fisher Scientific, Dreieich, Germany) using GP5+/6+ primers detecting the L1 gene (Eurofins MWG Operon, Ebersberg, Germany) (forward primer: 50-TTTGTTACTGTGGTAGATACTAC-30, reverse primer: 50-GAAAAATAAACTGTAAATCATATTC-30; amplicon size: 142 bp) [21]. The b-globin gene served as quality control (forward primer: 50-CAGGTACGGCTGTCATCACTTAGA-30, reverse primer: 50-CATGGTGTCTGTTTGAGGTTGCTA-30; amplicon size: 185 bp) (Metabion International AG, Planegg-Martinsried, Germany) [22]. Two HPV-positive (UPCI SCC2 and UPCI SCC154) and two HPV-negative (Cal27 and Cal33) cell lines were included as controls. Reactions were carried out in triplicate with negative controls. Samples with a detectable b-globin PCR product (Ct-value < 35) were considered HPV-negative if no HPV amplification product was detectable. Samples with a specific HPV PCR amplicon, as verified in melt curve analysis were considered HPV DNA positive.

A tumour specimen was finally classified as HPV-positive if it was positive for both p16^INK4a^ IHC and HPV DNA status assessed by GP 5+/6+ q-PCR (see Smeets et al., 2007) [23].

#### 2.3.2. HPV E6/E7 RNA Status

Whole RNA sequencing on LMU-KKG tumour specimens was performed as described previously [24] and in the paragraph ‘Whole RNA sequencing’. Trimmed reads were mapped to the human genome (GRCh38 version 87) and E6 and E7 gene sequences of 20 HNSCC associated HPV variants (HPV6, HPV11, HPV13, HPV16, HPV18, HPV31, HPV32, HPV33, HPV35, HPV38, HPV39, HPV45, HPV51, HPV52, HPV56, HPV58, HPV59, HPV66, HPV68, HPV69) [25], and quantified using the Salmon pseudo-aligner (v0.9.1) [26]. Quality of raw and mapped reads were assessed using FastQC (v0.11.6), and the resulting reports were summarized using MultiQC (v1.6). For further analysis transcripts per million (TPM) were calculated according to Patro et al., 2017 [26]. Cases with mRNA expression of E6 and E7 of the same HPV variant were considered to be HPV E6/E7 RNA positive.

### 2.4. HPV Characterisation—DKTK-ROG Cohort

#### 2.4.1. p16/HPV DNA Status

p16 and HPV DNA status for DKTK-ROG specimens was provided by the DKTK-ROG consortium [16]. A tumour specimen was classified as p16/HPV DNA positive if it was positive for both p16^INK4a^ IHC (CINtec Histology Kit, Roche mtm laboratories AG, Basel, Switzerland) and HPV DNA status assessed using the LCD-Array HPV 3.5 kit (CHIPRON GmbH, Berlin, Germany), as described previously [16].

#### 2.4.2. HPV16 E6/E7 RNA Status

HPV16 E6/E7 RNA status for DKTK-ROG specimens was provided by the DKTK-ROG consortium [27,28]. HPV16 E6/E7 RNA expression levels were determined by nanoString analysis (nanoString Technologies, Seattle, WA, USA) as described previously [27,28]. Tumours positive for both, HPV E6 and HPV E7 mRNA expression were classified as HPV16 E6/E7 RNA-positive.

### 2.5. miRNA Profiling

miRNA-microarray profiling using SurePrint G3 8x60K Human miRNA Microarrays (AMADID 70156; Agilent Technologies, Santa Clara, CA, USA) was performed as described previously [11]. Scanned miRNA Microarray intensities were written into text files before import into the R statistical platform. Pre-processing of the miRNA microarray data was conducted as described previously [11]. In brief, text files were imported into R using the AgiMicroRna Bioconductor package. After quality filtering using Agilent quality filters and removing non-human miRNAs from the data set, scanned intensities were background corrected, quantile normalized. The resulting expressions were log2-transformed after averaging signals determining expression of the same miRNA. MiRNA data were deposited at Gene Expression Omnibus under GSE175509.

### 2.6. Differential miRNA Expression Analysis

Differentially expressed miRNA between HPV-positive and HPV-negative tumours (DKTK-ROG and LMU-KKG combined) were determined using the Limma approach [29]. Significantly different expression was accepted for |log2 fold change > 0.5| with a false discovery rate (FDR) of <0.05.

### 2.7. Machine Learning and Performance Testing

We built a generalized-linear model with HPV status (ground truth: p16/HPV DNA status) as the response variable, and a signature of miRNAs as explanatory variables. miRNAs with a *p*-value < 0.05 in univariate association testing (Mann-Whitney test) were used as priors in feature selection by penalized lasso regression. Overfitting was addressed by 100 repeated 8-fold cross-validation using ROC-AUC as the summary metric for model optimization, while lambda was varied per iteration between 0.001 and 0.3 in steps of 0.001. The miRNA expression matrix of the DKTK-ROG cohort (*n* = 128) was used as a training data set with p16/HPV DNA status as ground truth. The prediction performance of the model was tested in the LMU-KKG miRNA-expression data set (*n* = 101). To assign patients of the discovery and validation sets to the HPV-negative and HPV-positive groups, the logit function was applied on the scalar product of signature-miRNA expressions and the appropriate model coefficients for the calculation of the prediction probability score per patient. Tumours were predicted as HPV-positive if the prediction probability score was >0.5, and as HPV-negative if ≤0.5. For the assessment of model performance, prediction accuracy, sensitivity, specificity, and ROC-AUC were used. All machine learning and performance assessment steps were performed using the caret R-package.

### 2.8. Clinical Endpoints

Overall survival was calculated from the start date of radiotherapy treatment to the date of death from any cause, locoregional control from the date of radiotherapy treatment start to the date of locoregional recurrence, and freedom from recurrence from the date of radiotherapy treatment start to the date of locoregional or distant recurrence. In the absence of an event, patients were censored at the date of the last follow-up visit (or the date of death).

### 2.9. Survival Analysis

Ground truth (p16/HPV DNA) and predicted HPV-status were tested for prognostic significance in the combined DKTK-ROG and LMU-KKG cohort for clinical endpoints overall survival, locoregional control and freedom from recurrence. A univariable Cox proportional-hazard model was fitted using the survival function as the response variable and the binary HPV-status as the explanatory variable. Log-rank test *p*-values < 0.05 were considered statistically significant. Hazard ratios (HR) with 95% confidence interval (CI) were reported.

### 2.10. Whole RNA Sequencing

Whole RNA sequencing on LMU-KKG tumour specimens was performed as described previously [24]: RNA integrity was determined using the Bioanalyzer Agilent RNA 6000 Nano chip (Agilent Technologies). The percentage of fragments >200 nucleotides (DV200) for each sample was calculated and samples were grouped into good (DV200 > 70%), medium (DV200 50–70%) and low quality (DV200 10–50%). RNA sequencing libraries were prepared with 500 ng (good quality) and 1000 ng (medium/low quality) total RNA using the TruSeq Stranded Total RNA Library Prep Gold with single-indexing following the manufacturer’s instructions protocol for high sample numbers (Illumina, Inc., San Diego, CA, USA). Initial RNA fragmentation time was adjusted to the respective RNA quality with 8 min (DV200 > 70%), 6 min (DV200 70–60%), 4 min (DV200 59–50), 0 min (DV200 < 49%). Quality and quantity of the libraries were evaluated using the Quanti-iT PicoGreen dsDNA Assay Kit (Thermo Fisher Scientific) and the Bioanalyzer High Sensitivity DNA Analysis Kit (Agilent Technologies). Library sequencing was performed on the Illumina HiSeq2000 platform. Trimmomatic (v0.36) was used to remove sequencing adapters from the raw reads, and trimmed reads were assigned to the human genome (GRCh38 version 87) and quantified with the pseudo-aligner salmon (v0.9.1). The quality of raw and mapped reads was evaluated with FastQC (v0.11.6), and the resulting reports were summarized with MultiQC (v1.6).

To characterize the functional impact of the identified miRNA-signature in HPV-positive and HPV-negative tumours, RNA-seq data from the following *n* = 30 LMU-KKG tumour specimens were included into the analyses: (1) *n* = 15 p16/HPV DNA-positive and 24-miRNA signature positive cases with available RNA-seq data and the top 15 highest prediction values; (2) *n* = 15 p16/HPV DNA-negative and 24-miRNA signature negative cases with available RNA-seq data and the top 15 lowest prediction values (Tables S4 and S6).

Processed RNA-seq data were deposited at our website (file created 26 May 2021): https://www.helmholtz-muenchen.de/fileadmin/ZYTO/other/GEx_HNSCC_HPV_miRNA_score_VST_250521.csv.

### 2.11. Differential Gene Expression Analysis

Differentially expressed genes between HPV-positive and HPV-negative tumours were determined by the approach as implemented in the DESeq2 R package while significance was accepted for |log2-fold change > 0.5| and FDR < 0.1 [30].

### 2.12. Cancer Signaling Pathway Analysis

The activity of 14 cancer-associated signaling pathways was inferred from the transcriptome data using the Bioconductor R package Progeny [31]. The z-scaled activity scores of a signaling pathway were calculated for each patient sample included in the dataset and subjected to a differential test between HPV-positive and HPV-negative groups using Mann-Whitney testing. Statistical significance was accepted for Benjamini-Hochberg FDR corrected *p*-values < 0.05 [32].

### 2.13. miRNA-mRNA Integration

To characterize the functional impact of the identified miRNA-signature in HPV-positive and HPV-negative tumours, we integrated the signature miRNA-expression with the mRNA-expression data of the appropriate groups.

Possible signature-miRNA-mRNA interactions revealed by seed sequence-based prediction were downloaded from DIANA, Miranda, PicTar and TargetScan using miRNAtap R function, as implemented in the SpidermiRdownload_miRNAprediction function of the SpidermiR R package (v1.26.0) [33]. The obtained possible interactions were tested for significance in the matched miRNA and mRNA expression data sets by determining the coefficients in Pearson correlation, mutual information and lasso regression as implemented in the SpidermiR R package [33]. From each of the three approaches, the intersection of the candidate interactions with the top 1000 coefficients was formed and tested for enrichment in gene sets of the Kyoto encyclopedia of genes and genomes as deposited at the Broad Institute MSigDB (http://www.gsea-msigdb.org, accessed on 17 January 2022) using the enrichKEGG function of the clusterProfiler R package. Enrichments were considered statistically significant if resulting false-discovery controlled *p*-values were <0.05.

## 3. Results

An overview of the clinicopathologic characteristics of all HNSCC patients investigated is provided in Table 1. The median follow-up times were 4.9 years (IQR 3.7–5.3) for the multicentre DKTK-ROG (*n* = 128; *n* = 38 HPV-positive, *n* = 90 HPV-negative) and 5.2 years (IQR 4.0–6.3) for the single-centre LMU-KKG (*n* = 101; *n* = 23 HPV-positive, *n* = 78 HPV-negative) cohorts. All patients were treated by adjuvant radio(chemo)therapy. HPV-positive cases were mainly oropharyngeal tumours (DKTK-ROG 34/38; LMU-KKG 20/23; Table 1).

**Figure 1:**
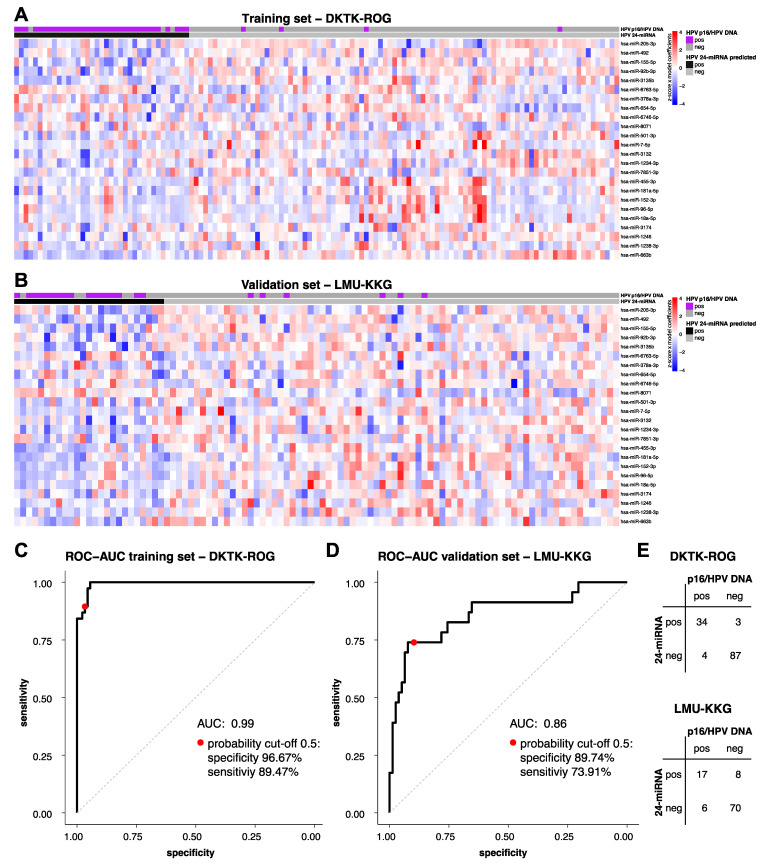
HPV-status predicted by the 24-miRNA signature. Heat map colours indicate scaled miRNA log2 expression values multiplied by the model coefficients from low (blue) to high (red) on a scale of −4 to 4 for each of the 24 signature miRNAs (**A**) in the training DKTK-ROG and (**B**) in the LMU-KKG validation set. HPV-status according to p16/HPV DNA and the 24-miRNA signature is indicated on a case-by-case basis. Performance of the 24-miRNA signature: sensitivity-derived and specificity-derived ROC-AUC for HPV-status prediction (**C**) in the DKTK-ROG training set and (**D**) in the LMU-KKG validation set. The AUC and the sensitivity and specificity rates at a probability cut-off of 0.5 are given. (**E**) Confusion matrix reporting the number of 24-miRNA signature predicted and p16/HPV DNA-positive and -negative HNSCC cases in the DKTK-ROG (top) and the LMU-KKG set (bottom).

Global miRNA expression profiling in *n* = 229 HNSCC specimens identified a total of 1031 expressed miRNAs (Figure S1) and *n* = 55 differentially expressed miRNAs (FDR < 0.05) in HPV-positive (*n* = 61) versus HPV-negative tumours (*n* = 168), with 37 down-regulated and 18 up-regulated miRNAs (Table S1 and Figure S2). A linear regression model predicting HPV status was trained irrespectively of tumour localisation in the DKTK-ROG miRNA data, and tested in the LMU-KKG data. The model obtained included 24 miRNAs (Table S2) and predicted HPV-status with 94.53% accuracy (AUC: 0.99; 96.67% specificity, 89.47% sensitivity) in DKTK-ROG and 86.14% accuracy (AUC: 0.86; 89.74% specificity, 73.91% sensitivity) in the LMU-KKG set (Figure 1, Tables S3 and S4). Another model trained on oropharyngeal tumours only comprised 18 miRNAs (11/18 overlapping with the 24-miRNA signature) and predicted p16/HPV DNA status with lower accuracy (DKTK-ROG: 88.28%; LMU-KKG: 82.18%) compared to the 24-miRNA signature.

**Figure 2:**
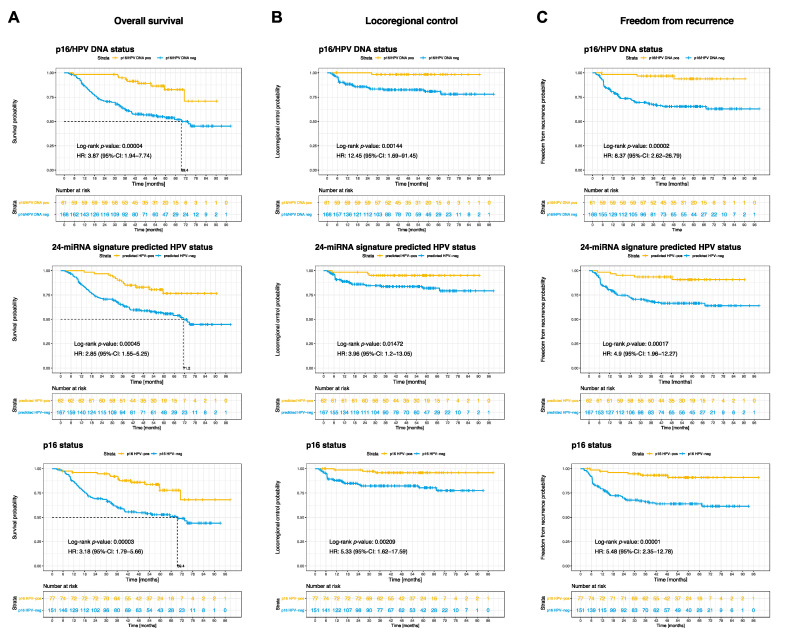
Prognostic value of the 24-miRNA signature and HPV-status. Kaplan-Meier curves for the endpoints (**A**) overall survival, (**B**) locoregional control and (**C**) freedom from recurrence stratified according to p16/HPV DNA status (top), 24-miRNA signature predicted HPV-status (middle), and p16 status (bottom). *p*-values are derived by log-rank test. Hazard ratios (HR) with 95% confidence intervals (CI) are given.

The prognostic value of HPV status predicted by the 24-miRNA signature was comparable to that of clinically used p16^INK4A^-typing and combined p16/HPV DNA status. HPV-stratified patient subgroups differed significantly in overall survival (OS), locoregional control, and freedom from recurrence (Figure 2).

**Figure 3:**
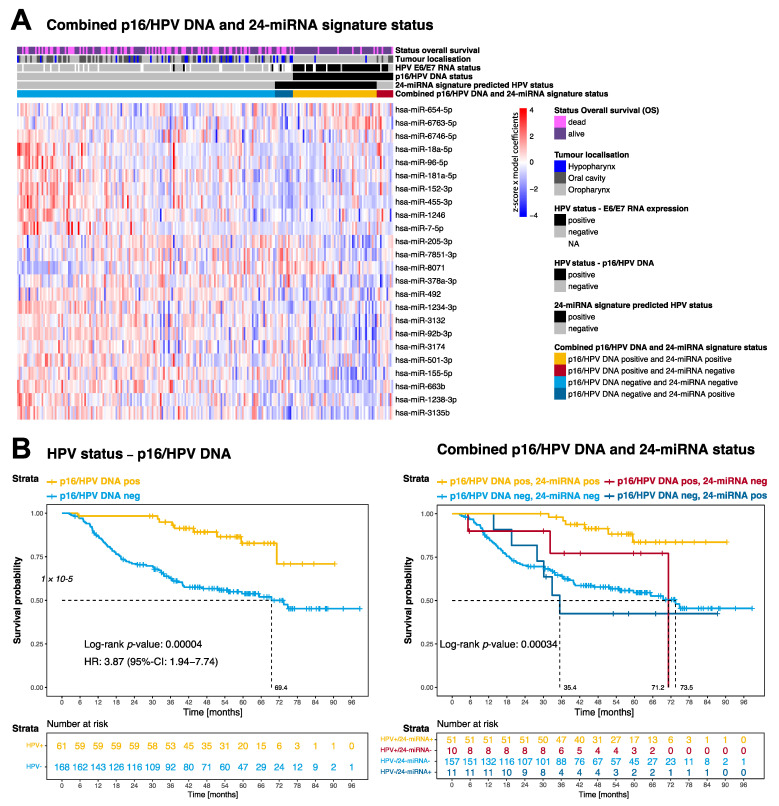
Combined p16/HPV DNA and 24-miRNA signature status. (**A**) Heat map colours indicate scaled miRNA log2 expression values multiplied by the model coefficients from low (blue) to high (red) on a scale of −4 to 4 for each of the 24 signature miRNAs. Information on overall survival status, tumour localisation, E6/E7 RNA expression HPV status, p16/HPV DNA status (ground truth), 24-miRNA signature predicted HPV status and combined p16/ HPV DNA and 24-miRNA signature status are indicated on a case-by-case basis. (**B**) Kaplan–Meier curves for the endpoint overall survival according to p16/HPV DNA status (left) and combined p16/HPV DNA and 24-miRNA signature status (right). *p*-values are derived by log-rank test. Hazard ratios (HR) with 95% confidence intervals (CI) and median overall survival times are given.

Sub-stratification of patients by combining p16/HPV DNA and 24-miRNA signature status (Figure 3) allowed the identification of an HPV-positive patient subgroup with impaired OS (*p* = 0.00034; Figure 3B).

In the subgroup of p16/HPV DNA-positive HNSCC (*n* = 61), a trend towards poorer OS (HR 3.44, 95%-CI 0.85–13.8; *p* = 0.065) was seen for cases (*n* = 10) predicted HPV-negative by the 24-miRNA signature compared to HPV-positive predicted cases (*n* = 51; Figure S3A). Five out of ten (50%) p16/HPV DNA-positive but 24-miRNA signature-negative HNSCCs were oral cavity tumours (Table S5). There was no association with smoking status (*p* = 0.44). On the RNA expression level, 6/10 were E6/E7 HPV positive (*n* = 4 HPV16, *n* = 1 HPV33, *n* = 1 HPV68, *n* = 1 NA), and *n* = 3 HPV16 E6/E7 negative DKTK-ROG cases might have involved another HPV subtype other than HPV16 (Tables S3 and S4). The HPV E6/E7 RNA expression data substantiate the p16/HPV DNA characterisation, and thus also the true HPV association of the subgroup of p16/HPV DNA-positive but 24-miRNA signature-negative HNSCCs with poorer overall survival.

In the subgroup of p16/HPV DNA-negative tumours (*n* = 168), 24-miRNA-positive (*n* = 11; 6.5%; Table S5) and 24-miRNA-negative cases (*n* = 157; 93.5%) did not differ regarding OS (Figure S3B).

**Figure 4:**
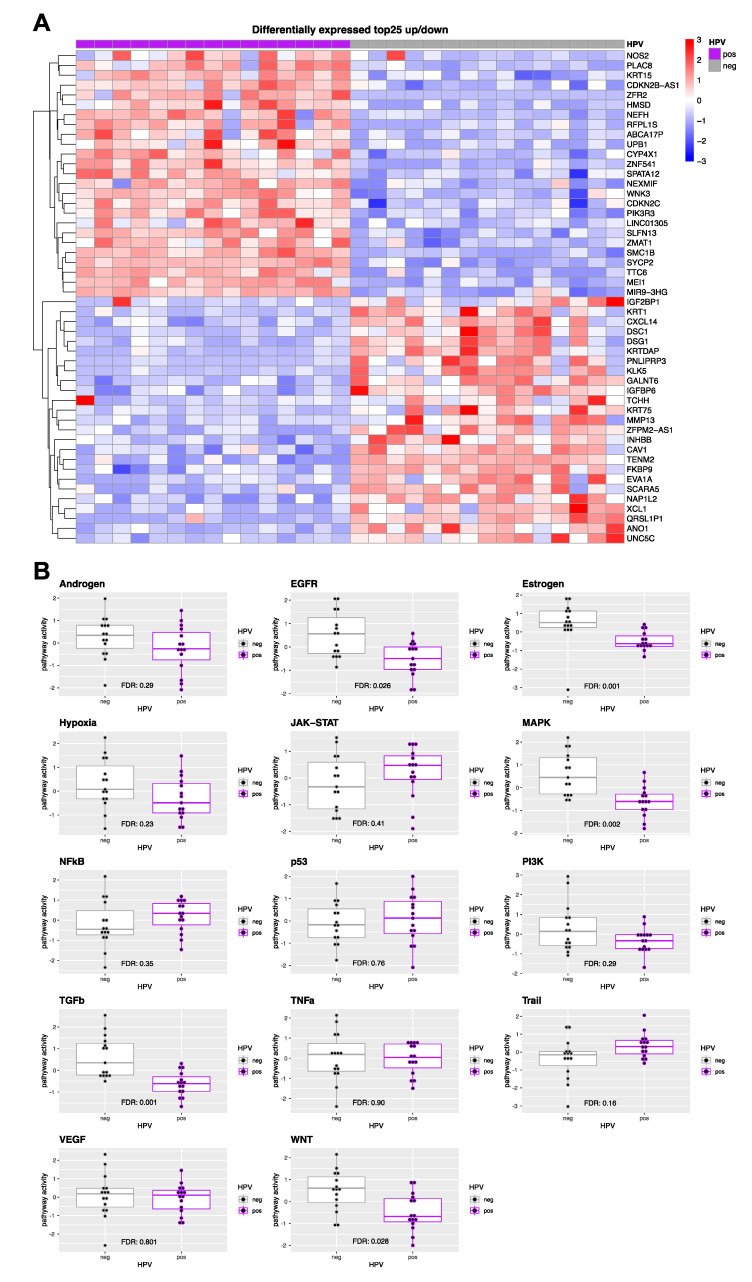
Differentially expressed genes and signaling pathway activity in 24-miRNA signature predicted HPV-positive versus HPV-negative HNSCC. (**A**) Heat map top 25 up-/down-regulated differentially expressed genes (adjusted *p*-value < 0.1 and |log2 fold change| > 0.5) in 24-miRNA signature positive and p16/HPV DNA-positive (*n* = 15) versus 24-miRNA signature negative and p16/HPV DNA-negative (*n* = 15) HNSCC of the LMU-KKG cohort. Heat map colours indicate mRNA expression z-scores on a scale of −3 to 3. (**B**) Boxplots illustrate signaling pathway activity derived by progeny analysis of differentially expressed genes (*n* = 658) in 24-miRNA-positive/HPV-positive (*n* = 15) and 24-miRNA-negative/HPV-negative (*n* = 15) HNSCC. Interquartile ranges with median centre lines are shown. FDR adjusted *p*-values are given.

To obtain insights into the biological regulatory function of the signature miRNAs, RNA-seq analysis was performed, including *n* = 15 p16/HPV DNA-/24-miRNA-positive and *n* = 15 p16/HPV DNA-/24-miRNA-negative LMU-KKG cases with the highest or lowest prediction scores, respectively (Tables S4 and S6). *n* = 658 genes were significantly differentially expressed (FDR < 0.1), with 452 down-regulated and 206 up-regulated genes in 24-miRNA-/HPV-positive versus 24-miRNA-/HPV-negative tumours (Table S7 and Figure 4A). *MAP kinase*, *Estrogen*, *EGFR*, *TGFbeta* and *WNT* signaling activity was significantly decreased in HPV-positive compared to HPV-negative tumours (Figure 4B).

**Figure 5:**
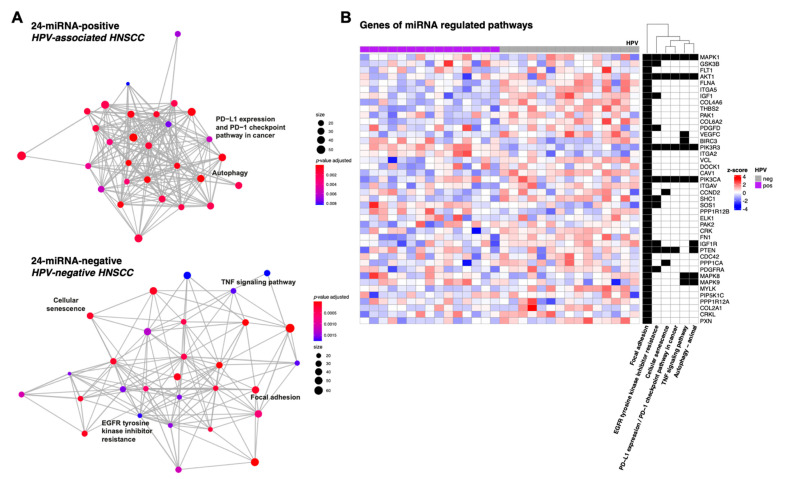
Pathway enrichment of 24 signature miRNA-mRNA interactions. (**A**) Enrichment maps with significantly enriched KEGG pathways (adjusted *p*-values < 0.05) of the top 1000 miRNA-mRNA interactions. Information on gene count/size (i.e., the number of genes enriched in a KEGG pathway) is given. Pathways occurring exclusively in 24-miRNA-positive/HPV-associated (upper panel) or 24-miRNA-negative/HPV-negative tumours (lower panel) are labelled. (**B**) Heatmap including genes of identified significantly enriched pathways exclusively associated with miRNA-mRNA interactions in 24-miRNA-positive/HPV-associated and 24-miRNA-negative/HPV-negative HNSCC. Heat map colours indicate mRNA expression z-scores on a scale of −4 to 4.

In a next step, miRNA-mRNA interactions calculated separately for 24-miRNA-/HPV-positive and 24-miRNA-/HPV-negative tumours. were subjected to KEGG pathway enrichment analysis. Most significantly enriched pathways were overlapping in the HPV-positive and HPV-negative groups. PD−L1 expression and PD−1 checkpoint pathway in cancer, autophagy was significantly enriched exclusively in HPV-positive and TNF signaling pathway, cellular senescence, focal adhesion, EGFR tyrosine kinase inhibitor resistance exclusively in HPV-negative tumours (Figure S4 and Figure 5).

In addition, genes likely to be regulated by the signature miRNAs and belonging to the signaling pathways that showed differential activity between HPV-positive and HPV-negative cases (*MAP kinase*, *Estrogen*, *EGFR*, *TGFbeta* and *WNT* signaling pathway; Figure 4B) were identified by matching the identified statistically significant miRNA-gene interactions with pathway gene sets from the Reactome database (Table S8).

## 4. Discussion

Here, we report a 24-miRNA signature that predicts HPV-status in HNSCC. The signature was identified in a multicentre miRNA dataset and validated in an independent single-centre dataset. In a simulation study, this strategy was shown to minimize the prediction error, leading to an increased chance for generalizability of the prediction model [34]. Both HNSCC cohorts comprised patients treated by adjuvant radio(chemo)therapy. Largely the same HPV-negative patients of a study in which we identified a 5-miRNA signature predicting recurrence and survival in HPV-negative HNSCC were included in the present study [11]. To the best of our knowledge, this is the first HPV-predicting miRNA signature validated in an independent dataset. The distributions of clinicopathological features between HPV-positive and HPV-negative HNSCC patients were the same in and between both cohorts. Consistent with published evidence, most HPV-positive tumours were of oropharyngeal origin (89%) [25]. To exclude cases with increased p16 expression by non-viral mechanisms, and to focus on the molecular biology of HPV-induced carcinogenesis, only tumours that were both p16^INK4A^ and HPV DNA-positive were considered HPV-positive [23]. We deliberately included HPV-positive HNSCCs of all tumour sites as prior studies on HPV-positivity in non-oropharyngeal tumours are divergent and mostly underpowered [35].

In the clinic, HPV-positive tumours are considered as a distinct entity, which is also reflected at different molecular levels including the miRNA level [1,2]. Previous studies have identified several deregulated miRNAs in HNSCC, some of which were also associated with HPV-status of tumours. Although the overlap of miRNAs from different HNSCC studies is generally relatively small, 12 of the differentially expressed miRNAs between HPV-positive and HPV-negative tumours (Table S1) and five signature miRNAs (hsa-miR-155-5p, hsa-miR-181a-5p, hsa-miR-18a-5p, hsa-miR-378a-3p, hsa-miR-455-3p) were previously reported to be HPV-associated [1,5,6,7,8,9,10,12]. The analysis of bulk samples does not allow differentiation between tumour and stromal miRNA sources (e.g., infiltrated lymphocytes), and could be a cause for the rather small differences in detected miRNA levels [36]. Nevertheless, the miRNA analyses enabled the identification of a 24-miRNA signature that, in addition to miRNA expression differences in tumour cells, could also be determined by those of the tumour microenvironment of HPV-positive and HPV-negative patients. The 24-miRNA signature not only allowed the prediction of the clinically applied p16/HPV DNA status with 86% accuracy, but stratified patients regarding all clinical endpoints analysed. Combining the 24-miRNA signature with p16/HPV DNA status even improved risk stratification of HPV-positive and HPV-negative tumours and allowed the identification of an HPV-associated patient subgroup with impaired OS. The trend towards poorer OS for 24-miRNA-negative tumours within the group of p16/HPV DNA-positive HNSCC compared to p16/HPV DNA-positive tumours also predicted to be HPV-positive by the 24-miRNA signature, further suggests distinct molecular evolutions of tumours. Strikingly, 50% of the p16/HPV DNA-positive/24-miRNA-negative cases were oral cavity tumours and, according to the E6/E7 RNA expression data, 20% involved HPV subtypes other than HPV16 (HPV33/HPV68), indicating the influence of tumour localisation and HPV variant on 24-miRNA signature expression patterns. In contrast, all p16/HPV DNA-positive tumours reported to originate from the hypopharynx were also predicted to be HPV-positive by the 24-miRNA signature, suggesting greater molecular similarities at the miRNA level between HPV-associated hypopharyngeal and oropharyngeal tumours. However, these conclusions remain speculative due to the small number of non-oropharyngeal tumours in our study. Previous studies on non-oropharyngeal HPV-associated HNSCC were also limited by small case numbers and were inconclusive regarding prognosis. Few studies have shown improved outcomes for non-oropharyngeal HPV-associated tumours, while even worse outcomes have been reported for patients with HPV-positive oral cavity tumours, consistent with our observations [35]. For oropharyngeal HNSCC, patient subgroups with different risk of death according to the HPV status, tobacco smoking, and cancer stage were demonstrated [20]. HPV-positive tumours associated with tobacco or alcohol are, in part, considered a separate entity, distinct from HPV-positive HNSCC without these classical risk factors. Among the p16/HPV DNA-positive tumours in our study, there was no association of the 24-miRNA-negative subgroup, which tended to have poorer survival, with alcohol or smoking.

In HPV-negative HNSCC, we did not detect differences in overall survival between subgroups defined by the 24-miRNA signature in this study, as was the case in a previous study of HPV-negative HNSCC in which we identified a 5-miRNA signature predictive of recurrence and survival and identified HPV-negative HNSCC subgroups with different prognosis [11].

miRNAs act as important post-transcriptional integrative regulators and interfere, due to the high promiscuity of a single miRNA to bind multiple mRNAs, with multiple signaling pathways, making it difficult to infer clear biological functions. However, using the transcriptome data for the analysis of differential signaling activity and integration with miRNA data in HPV-positive and HPV-negative tumours, we gained insights into the biological regulatory function of the 24-miRNA signature. Our findings are further strengthened by the fact that the functional assignment of the signature miRNAs suggests an important post-transcriptional integrative role in signaling pathways frequently deregulated in HNSCC, which have also been associated with the differential molecular biology of HPV-positive and HPV-negative tumours. As such, *TGFbeta* signaling activity has been shown to be decreased in HPV-positive HNSCC as a result of HPV infection, increasing sensitivity to radiation and chemotherapy by homologous recombination repair deficiency [37]. Stronger *WNT* signaling activation was suggested for HPV-negative patients consuming tobacco [38], also supported by our data. miRNA-mRNA interactions in 24-miRNA-/HPV-negative HNSCC showed an association with *EGFR* tyrosine kinase inhibitor resistance. The clinical benefit of the approved EGFR targeting monoclonal antibody Cetuximab is limited; with advanced disease status, resistance development and pleiotropic cellular functions of the EGFR pathway playing a role [3]. Furthermore, de-escalation trials in HPV-associated HNSCC (RTOG 1016, De-ESCALaTE), in which standard cisplatin regimen was replaced by cetuximab concurrent with radiation, showed inferior tumour control and survival [39]. The significantly decreased *EGFR* signaling pathway activity observed in 24-miRNA-/HPV-positive patients in our study might be a contributing factor to the unexpected trial results. The EGFR signaling pathway is a complex network that largely overlaps with other signaling pathways. For instance, EGFR can activate the *MAPK* signaling pathway, for which we have also demonstrated higher activity in 24-miRNA signature-/HPV-negative HNSCC. miRNA-mRNA interactions identified a number of commonly enriched pathways, as well as pathways exclusively associated with 24-miRNA signature-/HPV-negative HNSCC: the *TNF* signaling pathway, for which a promoting effect on lymph angiogenesis in HNSCC was suggested [40]; cellular senescence, previously shown to be involved in radioresistance and of therapeutic relevance in HNSCC [41,42]; and focal adhesion, involved in progression of HPV-negative HNSCC [43]. In addition, we demonstrated exclusive association of miRNA-mRNA interactions in 24-miRNA signature-/HPV-positive HNSCC with *PD-L1* expression and *PD*-1 checkpoint pathway in cancer. Increased PD-L1 expression and an immunologically active tumour microenvironment has been demonstrated for HPV-positive HNSCC, suggesting immunotherapy as a promising treatment option [44,45]. While the immune checkpoint inhibitor trials KEYNOTE-012 and KEYNOTE-048 reported better response rates and more likely benefit in p16-positive compared to p16-negative patients, other trials, such as KEYNOTE-040, showed a greater survival benefit for HPV-negative patients [46,47]. Overall, both HPV-positive and HPV-negative patients seem to benefit from immune checkpoint inhibitors. More studies are underway to investigate whether the treatment of HPV-positive HNSCC with immunotherapy is a potential lower cytotoxic alternative therapy.

## 5. Conclusions

In conclusion, the 24-miRNA signature accurately predicts HPV-status and, within HPV-positive HNSCC, even better reflects prognosis and molecular biology compared to HPV-typing using p16/HPV DNA status alone. Future clinical decision-making on treatment de-escalation in HPV-positive HNSCC might consider 24-miRNA signature classification combined with p16/HPV DNA status. Specifically, deregulated signaling pathways in HPV-positive or HPV-negative HNSCC identified by 24-miRNA signature-mRNA integration enable a deeper molecular understanding of both tumour entities. Moreover, the functional assignment of miRNAs suggests an important post-transcriptional integrative role in key deregulated pathways in HNSCC.

## Figures and Tables

**Table 1:** Clinicopathological HNSCC patient characteristics.

Parameter	DKTK-ROG				LMU-KKG			
	HPV-Negative (*n* = 90)	HPV-Positive (*n* = 38)	Total (*n* = 128)	*p*-Value	HPV-Negative (*n* = 78)	HPV-Positive (*n* = 23)	Total (*n* = 101)	*p*-Value
**Age (years)**				0.269				0.740
<45	8 (8.9%)	3 (7.9%)	11 (8.6%)		3 (3.8%)	1 (4.3%)	4 (4.0%)	
45–54	29 (32.2%)	8 (21.1%)	37 (28.9%)		17 (21.8%)	6 (26.1%)	23 (22.8%)	
55–64	36 (40.0%)	14 (36.8%)	50 (39.1%)		28 (35.9%)	9 (39.1%)	37 (36.6%)	
65–74	17 (18.9%)	13 (34.2%)	30 (23.4%)		27 (34.6%)	5 (21.7%)	32 (31.7%)	
>75					3 (3.8%)	2 (8.7%)	5 (5.0%)	
**Sex**				0.778				0.709
Male	73 (81.1%)	30 (78.9%)	103 (80.5%)		51 (65.4%)	16 (69.6%)	67 (66.3%)	
Female	17 (18.9%)	8 (21.1%)	25 (19.5%)		27 (34.6%)	7 (30.4%)	34 (33.7%)	
**Tumour localization**				<0.001				0.001
Hypopharynx	14 (15.6%)	1 (2.6%)	15 (11.7%)		16 (20.5%)	1 (4.3%)	17 (16.8%)	
Oral cavity	33 (36.7%)	3 (7.9%)	36 (28.1%)		28 (35.9%)	2 (8.7%)	30 (29.7%)	
Oropharynx	43 (47.8%)	34 (89.5%)	77 (60.2%)		34 (43.6%)	20 (87.0%)	54 (53.5%)	
**UICC TNM stage**				0.221				0.773
I					2 (2.6%)	0 (0.0%)	2 (2.0%)	
II	4 (4.4%)	2 (5.3%)	6 (4.7%)		7 (9.0%)	2 (8.7%)	9 (8.9%)	
III	15 (16.7%)	2 (5.3%)	17 (13.3%)		22 (28.2%)	5 (21.7%)	27 (26.7%)	
IV	71 (78.9%)	34 (89.5%)	105 (82.0%)		47 (60.3%)	16 (69.6%)	63 (62.4%)	
**TNM T stage**				0.601				0.834
T1	13 (14.4%)	4 (10.5%)	17 (13.3%)		17 (21.8%)	5 (21.7%)	22 (21.8%)	
T2	38 (42.2%)	21 (55.3%)	59 (46.1%)		31 (39.7%)	11 (47.8%)	42 (41.6%)	
T3	22 (24.4%)	7 (18.4%)	29 (22.7%)		18 (23.1%)	5 (21.7%)	23 (22.8%)	
T4	17 (18.9%)	6 (15.8%)	23 (18.0%)		12 (15.4%)	2 (8.7%)	14 (13.9%)	
**TNM N** **stage**				0.528				0.234
N0	12 (13.3%)	3 (7.9%)	15 (11.7%)		20 (25.6%)	7 (30.4%)	27 (26.7%)	
N1	10 (11.1%)	4 (10.5%)	14 (10.9%)		21 (26.9%)	2 (8.7%)	23 (22.8%)	
N2	59 (65.6%)	24 (63.2%)	83 (64.8%)		35 (44.9%)	14 (60.9%)	49 (48.5%)	
N3	9 (10.0%)	7 (18.4%)	16 (12.5%)		2 (2.6%)	0 (0.0%)	2 (2.0%)	
**Lymphovascular invasion (LVI)**				0.372				0.194
0	46 (63.9%)	24 (72.7%)	70 (66.7%)		52 (77.6%)	14 (63.6%)	66 (74.2%)	
1	26 (36.1%)	9 (27.3%)	35 (33.3%)		15 (22.4%)	8 (36.4%)	23 (25.8%)	
Missing information	18	5	23		11	1	12	
**Venous tumour invasion (VTI)**				0.244				0.331
0	65 (90.3%)	31 (96.9%)	96 (92.3%)		66 (95.7%)	21 (100.0%)	87 (96.7%)	
1	7 (9.7%)	1 (3.1%)	8 (7.7%)		3 (4.3%)	0 (0.0%)	3 (3.3%)	
Missing information	18	6	24		9	2	11	
**Perineural invasion (PNI)**								0.485
0	0 (0.0%)	0 (0.0%)	0 (0.0%)		37 (74.0%)	14 (82.4%)	51 (76.1%)	
1	0 (0.0%)	0 (0.0%)	0 (0.0%)		13 (26.0%)	3 (17.6%)	16 (23.9%)	
Missing information	90	38	128		28	6	34	
**Resection margin status**				0.157				0.361
0	47 (52.2%)	25 (65.8%)	72 (56.2%)		59 (77.6%)	15 (65.2%)	74 (74.7%)	
1	43 (47.8%)	13 (34.2%)	56 (43.8%)		16 (21.1%)	8 (34.8%)	24 (24.2%)	
2					1 (1.3%)	0 (0.0%)	1 (1.0%)	
Missing information					2	0	2	
**ECE**				0.595				0.828
Not applicable (N0)	12 (13.3%)	3 (7.9%)	15 (11.7%)		20 (26.0%)	7 (30.4%)	27 (27.0%)	
No	33 (36.7%)	13 (34.2%)	46 (35.9%)		32 (41.6%)	10 (43.5%)	42 (42.0%)	
Yes	45 (50.0%)	22 (57.9%)	67 (52.3%)		25 (32.5%)	6 (26.1%)	31 (31.0%)	
Missing information	0	0	0		1	0	1	
**Smoking status**				0.050				0.078
Missing information	30	13	43		18 (23.1%)	5 (21.7%)	23 (22.8%)	
Nonsmoker	5 (8.3%)	6 (24.0%)	11 (12.9%)		3 (3.8%)	4 (17.4%)	7 (6.9%)	
Smoker	55 (91.7%)	19 (76.0%)	74 (87.1%)		57 (73.1%)	14 (60.9%)	71 (70.3%)	
**Simultaneous chemotherapy**								0.553
Yes	90 (100.0%)	38 (100.0%)	128 (100.0%)		49 (62.8%)	16 (69.6%)	65 (64.4%)	
No	0 (0.0%)	0 (0.0%)	0 (0.0%)		29 (37.2%)	7 (30.4%)	36 (35.6%)	

## Data Availability

Processed RNA-seq data from *n* = 30 LMU-KKG HNSCC specimens have been deposited at our website (file created 26 May 2021): https://www.helmholtz-muenchen.de/fileadmin/ZYTO/other/GEx_HNSCC_HPV_miRNA_score_VST_250521.csv.

## Supplementary Materials

The following supporting information can be downloaded at: https://www.mdpi.com/article/10.3390/cancers14153745/s1, Table S1: Differentially expressed miRNAs (*n* = 55; FDR adjusted *p*-value < 0.05 and |log2 fold change| > 0.5) in HPV-positive (*n* = 61) versus HPV-negative (*n* = 168) HNSCC; Table S2: 24 signature miRNAs with model coefficients; Table S3: DKTK-ROG cohort–HPV status (p16/HPV DNA, E6/E7 RNA) and 24miRNA prediction; Table S4: LMU-KKG cohort–HPV status (p16/HPV DNA, E6/E7 RNA) and 24miRNA prediction; Table S5: Clinicopathological characteristics of HNSCC patient subgroups p16/HPV DNA positive and 24-miRNA signature negative (*n* = 10) versus p16/HPV DNA negative and 24-miRNA signature positive (*n* = 11); Table S6: Clinicopathological characteristics of HNSCC patients included in RNA-seq analysis; Table S7: Differentially expressed genes (*n* = 658; FDR adjusted *p*-value < 0.1 and |log2 fold change| > 0.5) in 24-miRNA signature-positive/HPV-positive (*n* = 15) versus 24-miRNA signature-negative/HPV-negative (*n* = 15) HNSCC; Table S8: Genes likely to be regulated by the signature miRNAs and belonging to the signalling pathways that showed differential activity between HPV-positive and HPV-negative cases in Progeny analysis (MAP kinase, Estrogen, EGFR, TGFbeta and WNT signalling pathway); Figure S1: Unsupervised hierarchical clustering heatmap including all expressed miRNAs (*n* = 1031); Figure S2: Heatmap differentially expressed miRNAs (*n* = 55; adjusted *p*-value < 0.05 and |log2 fold change| > 0.5) in HPV-positive (*n* = 61) versus HPV-negative (*n* = 168) HNSCC (p16/HPV DNA status); Figure S3: Kaplan–Meier curves for the endpoint overall survival according to the 24-miRNA signature predicted HPV-status in p16/HPV DNA-positive and p16/HPV DNA-negative patients; Figure S4: Significantly enriched KEGG pathways (adjusted *p*-values < 0.05) of the top 1000 miRNA-mRNA interactions in 24-miRNA-positive/HPV-associated and 24-miRNA-negative/HPV-negative HNSCC.
