# Supplementary material for: Integration of p16/HPV DNA Status with a 24-miRNA-Defined Molecular Phenotype Improves Clinically Relevant Stratification of Head and Neck Cancer Patients
Source: Cancers (Basel). 2022 Jul 31;14(15):3745. doi: 10.3390/cancers14153745 (PMC9367561; doi:10.3390/cancers14153745)

Supplementary Materials

Supplementary Tables

Supplementary Table S1

Differentially expressed miRNAs (n = 55; FDR adjusted *p*-value < 0.05 and |log2 fold change| > 0.5) in HPV-positive (n = 61) versus HPV-negative (n = 168) HNSCC. *HPV association in HNSCC published before.

miRNA	log2 fold change	*p*-value	Adjusted *p*-value	literature
hsa-miR-663b	-0.972832246	5.40377E-11	5.57129E-08	
hsa-miR-155-5p	0.762530316	2.72575E-07	0.000140512	*
hsa-miR-127-3p	-0.719700868	1.21782E-06	0.000418525	*
hsa-miR-376c-3p	-0.682914513	4.13084E-06	0.001014637	
hsa-miR-376a-3p	-0.673742353	5.54998E-06	0.001014637	
hsa-miR-663a	-0.671803104	5.90477E-06	0.001014637	
hsa-miR-92b-3p	0.649644478	1.18479E-05	0.001721624	
hsa-miR-193b-3p	-0.645349785	1.35264E-05	0.001721624	*
hsa-miR-3135b	0.639594891	1.61339E-05	0.001721624	
hsa-miR-492	0.638466326	1.66986E-05	0.001721624	
hsa-miR-214-3p	-0.624889506	2.5149E-05	0.002268273	
hsa-miR-934	-0.623260986	2.64008E-05	0.002268273	
hsa-miR-381-3p	-0.613610193	3.51251E-05	0.00271845	*
hsa-miR-3132	-0.611917078	3.6914E-05	0.00271845	
hsa-miR-205-3p	0.609309853	3.98387E-05	0.002726837	
hsa-miR-564	-0.606940071	4.26867E-05	0.002726837	
hsa-miR-1260b	-0.603159078	4.76342E-05	0.002726837	
hsa-miR-4632-5p	-0.602291355	4.88439E-05	0.002726837	
hsa-miR-455-3p	-0.599781288	5.25095E-05	0.002726837	*
hsa-miR-3934-3p	-0.599525779	5.28969E-05	0.002726837	
hsa-miR-142-5p	0.59726996	5.64368E-05	0.002770779	*
hsa-miR-6763-5p	-0.592930186	6.38872E-05	0.002977638	
hsa-miR-5587-5p	-0.590686461	6.80952E-05	0.002977638	
hsa-miR-645	-0.58950462	7.04158E-05	0.002977638	
hsa-miR-3196	-0.588619487	7.22027E-05	0.002977638	
hsa-miR-1909-5p	0.586277731	7.71391E-05	0.00305886	
hsa-miR-99b-5p	-0.584504698	8.10871E-05	0.003096325	*
hsa-miR-6877-3p	0.582690334	8.53244E-05	0.003141765	
hsa-miR-874-3p	-0.578151747	9.68586E-05	0.003443491	
hsa-miR-361-3p	0.575337657	0.000104734	0.003599348	
hsa-miR-378d	0.574083127	0.000108436	0.003606365	
hsa-miR-648	-0.567958586	0.000128351	0.004135304	
hsa-miR-6791-5p	-0.562052574	0.000150781	0.004494932	
hsa-miR-4436b-5p	0.562041677	0.000150825	0.004494932	
hsa-miR-378a-3p	0.560887479	0.000155621	0.004494932	*
hsa-miR-3620-3p	0.559854013	0.000160036	0.004494932	
hsa-miR-3200-5p	-0.559560321	0.000161312	0.004494932	
hsa-miR-146a-5p	0.54961197	0.000210642	0.005709838	*
hsa-miR-3622b-5p	-0.548669336	0.000215988	0.005709838	
hsa-miR-206	-0.546023618	0.00023168	0.005971558	
hsa-miR-150-5p	0.539785988	0.000273013	0.006865269	*
hsa-miR-6507-5p	-0.53822334	0.0002844	0.006902366	
hsa-miR-708-5p	-0.537757779	0.000287878	0.006902366	
hsa-miR-3653-3p	0.534720892	0.000311559	0.007300391	
hsa-miR-6830-5p	-0.530135067	0.000350798	0.008037173	
hsa-miR-2277-3p	0.523910129	0.000411491	0.009222767	
hsa-miR-3689f	-0.521463089	0.000437931	0.009606534	
hsa-miR-195-3p	0.514564051	0.000521262	0.010914483	*
hsa-miR-4695-5p	-0.514530998	0.000521695	0.010914483	
hsa-miR-6829-5p	-0.513952939	0.000529315	0.010914483	
hsa-miR-1185-1-3p	-0.508571773	0.000605403	0.012238646	
hsa-miR-145-5p	-0.507197769	0.000626401	0.012419604	*
hsa-miR-1208	-0.505249037	0.000657345	0.012787229	
hsa-miR-614	-0.502834701	0.000697655	0.013320035	
hsa-miR-6126	-0.501412255	0.000722467	0.013542968	

Supplementary Table S2
24 signature miRNAs with model coefficients

miRNA	model coefficient
hsa-miR-663b	1.308121239
hsa-miR-1238-3p	0.835272594
hsa-miR-1246	0.724306076
hsa-miR-3174	0.514668701
hsa-miR-18a-5p	0.463953711
hsa-miR-96-5p	0.441121083
hsa-miR-152-3p	0.411084373
hsa-miR-181a-5p	0.26391495
hsa-miR-455-3p	0.222267731
hsa-miR-7851-3p	0.19008692
hsa-miR-1234-3p	0.137228246
hsa-miR-3132	0.116679027
hsa-miR-7-5p	0.096718214
hsa-miR-501-3p	0.087255656
hsa-miR-8071	0.042703364
hsa-miR-6746-5p	-0.181571535
hsa-miR-654-5p	-0.256513707
hsa-miR-378a-3p	-0.312449364
hsa-miR-6763-5p	-0.399115225
hsa-miR-3135b	-0.482922457
hsa-miR-92b-3p	-0.551388586
hsa-miR-155-5p	-0.559364753
hsa-miR-492	-0.897070347
hsa-miR-205-3p	-1.163273803

Supplementary Table S3
DKTK-ROG cohort – HPV status (p16/HPV DNA, E6/E7 RNA) and 24miRNA prediction

Patient ID	p16/HPV DNA	HPV_E6_E7	HPV16_E6	HPV16_E7	24miRNA predicted status	24miRNA prediction score
DKTKRO_114	pos	pos	expressed	expressed	pos	0.995000332
DKTKRO_87	pos	pos	expressed	expressed	pos	0.993958921
DKTKRO_100	pos	pos	expressed	expressed	pos	0.991786263
DKTKRO_102	pos	pos	expressed	expressed	pos	0.985265481
DKTKRO_113	pos	pos	expressed	expressed	pos	0.969770965
DKTKRO_109	pos	pos	expressed	expressed	pos	0.947392242
DKTKRO_118	pos	pos	expressed	expressed	pos	0.946842792
DKTKRO_124	pos	pos	expressed	expressed	pos	0.945148671
DKTKRO_108	pos	pos	expressed	expressed	pos	0.923011919
DKTKRO_123	pos	pos	expressed	expressed	pos	0.917520416
DKTKRO_39	pos	neg	not_expressed	not_expressed	pos	0.910511548
DKTKRO_92	pos	pos	expressed	expressed	pos	0.899740589
DKTKRO_89	pos	pos	expressed	expressed	pos	0.893047617
DKTKRO_117	pos	pos	expressed	expressed	pos	0.892687008
DKTKRO_96	pos	pos	expressed	expressed	pos	0.886615354
DKTKRO_86	pos	pos	expressed	expressed	pos	0.883425081
DKTKRO_97	pos	pos	expressed	expressed	pos	0.879398467
DKTKRO_106	pos	pos	expressed	expressed	pos	0.868768871
DKTKRO_111	pos	pos	expressed	expressed	pos	0.863697103
DKTKRO_127	pos	NA	NA	NA	pos	0.852157203
DKTKRO_88	pos	pos	expressed	expressed	pos	0.833737603
DKTKRO_99	pos	pos	expressed	expressed	pos	0.820295535
DKTKRO_126	pos	pos	expressed	expressed	pos	0.816785505
DKTKRO_95	pos	pos	expressed	expressed	pos	0.815048166
DKTKRO_105	pos	pos	expressed	expressed	pos	0.7883644
DKTKRO_125	pos	NA	NA	NA	pos	0.780614359
DKTKRO_112	pos	pos	expressed	expressed	pos	0.758567651
DKTKRO_104	pos	pos	expressed	expressed	pos	0.754003514
DKTKRO_101	pos	pos	expressed	expressed	pos	0.743742505
DKTKRO_120	pos	pos	expressed	expressed	pos	0.702105155
DKTKRO_93	pos	pos	expressed	expressed	pos	0.697976222
DKTKRO_122	pos	pos	expressed	expressed	pos	0.653592753
DKTKRO_82	neg	pos	expressed	expressed	pos	0.564083323
DKTKRO_76	neg	neg	not_expressed	not_expressed	pos	0.555125247
DKTKRO_110	pos	pos	expressed	expressed	pos	0.527667587
DKTKRO_04	neg	NA	NA	NA	pos	0.519804714
DKTKRO_98	pos	pos	expressed	expressed	pos	0.507927192
DKTKRO_84	neg	pos	expressed	expressed	neg	0.496458659
DKTKRO_91	pos	neg	not_expressed	not_expressed	neg	0.493963947
DKTKRO_107	pos	neg	not_expressed	not_expressed	neg	0.437042447
DKTKRO_94	pos	neg	not_expressed	not_expressed	neg	0.43643643
DKTKRO_06	neg	neg	not_expressed	not_expressed	neg	0.388811822
DKTKRO_121	pos	pos	expressed	expressed	neg	0.384918478
DKTKRO_33	neg	neg	not_expressed	not_expressed	neg	0.360544389
DKTKRO_23	neg	neg	not_expressed	not_expressed	neg	0.349532466
DKTKRO_25	neg	neg	not_expressed	not_expressed	neg	0.323130394
DKTKRO_29	neg	neg	not_expressed	not_expressed	neg	0.289341391
DKTKRO_81	neg	neg	not_expressed	not_expressed	neg	0.194636203
DKTKRO_19	neg	neg	not_expressed	not_expressed	neg	0.16841202
DKTKRO_37	neg	neg	not_expressed	not_expressed	neg	0.157300928
DKTKRO_24	neg	neg	not_expressed	not_expressed	neg	0.14388572
DKTKRO_28	neg	neg	not_expressed	not_expressed	neg	0.139872487

Patient ID	p16/HPV DNA	HPV_E6_E7	HPV16_E6	HPV16_E7	24miRNA predicted status	24miRNA prediction score
DKTKRO_69	neg	neg	not_expressed	not_expressed	neg	0.133894951
DKTKRO_40	neg	neg	not_expressed	not_expressed	neg	0.128646922
DKTKRO_11	neg	neg	not_expressed	not_expressed	neg	0.116570486
DKTKRO_75	neg	neg	not_expressed	not_expressed	neg	0.112820847
DKTKRO_48	neg	neg	not_expressed	not_expressed	neg	0.112053879
DKTKRO_45	neg	neg	not_expressed	not_expressed	neg	0.107490336
DKTKRO_66	neg	neg	not_expressed	not_expressed	neg	0.095951434
DKTKRO_03	neg	neg	not_expressed	not_expressed	neg	0.091153084
DKTKRO_62	neg	neg	not_expressed	not_expressed	neg	0.087728739
DKTKRO_30	neg	neg	not_expressed	not_expressed	neg	0.08618827
DKTKRO_115	neg	neg	not_expressed	not_expressed	neg	0.082725128
DKTKRO_70	neg	neg	not_expressed	not_expressed	neg	0.079399933
DKTKRO_52	neg	NA	NA	NA	neg	0.076532533
DKTKRO_59	neg	neg	not_expressed	not_expressed	neg	0.076249225
DKTKRO_73	neg	neg	not_expressed	not_expressed	neg	0.073600479
DKTKRO_13	neg	NA	NA	NA	neg	0.071980427
DKTKRO_63	neg	pos	expressed	expressed	neg	0.070415836
DKTKRO_85	neg	neg	not_expressed	not_expressed	neg	0.067884255
DKTKRO_42	neg	neg	not_expressed	not_expressed	neg	0.067682988
DKTKRO_41	neg	neg	not_expressed	not_expressed	neg	0.064656374
DKTKRO_21	neg	neg	not_expressed	not_expressed	neg	0.06053441
DKTKRO_58	neg	pos	expressed	expressed	neg	0.058669967
DKTKRO_16	neg	neg	not_expressed	not_expressed	neg	0.058571273
DKTKRO_119	neg	neg	not_expressed	not_expressed	neg	0.05368363
DKTKRO_07	neg	neg	not_expressed	not_expressed	neg	0.052046937
DKTKRO_71	neg	neg	not_expressed	not_expressed	neg	0.052037628
DKTKRO_27	neg	neg	expressed	not_expressed	neg	0.048294101
DKTKRO_14	neg	neg	not_expressed	not_expressed	neg	0.045984534
DKTKRO_12	neg	neg	not_expressed	not_expressed	neg	0.045701369
DKTKRO_36	neg	neg	not_expressed	not_expressed	neg	0.041057448
DKTKRO_60	neg	neg	not_expressed	not_expressed	neg	0.037965256
DKTKRO_18	neg	neg	not_expressed	not_expressed	neg	0.036653174
DKTKRO_22	neg	neg	not_expressed	not_expressed	neg	0.034317007
DKTKRO_15	neg	neg	not_expressed	not_expressed	neg	0.032062969
DKTKRO_53	neg	neg	not_expressed	not_expressed	neg	0.031764482
DKTKRO_55	neg	neg	not_expressed	not_expressed	neg	0.03153084
DKTKRO_79	neg	neg	not_expressed	not_expressed	neg	0.028872928
DKTKRO_09	neg	neg	not_expressed	not_expressed	neg	0.028799191
DKTKRO_68	neg	neg	not_expressed	not_expressed	neg	0.028497304
DKTKRO_51	neg	neg	not_expressed	not_expressed	neg	0.025225501
DKTKRO_10	neg	neg	not_expressed	not_expressed	neg	0.023363856
DKTKRO_80	neg	neg	not_expressed	not_expressed	neg	0.020575474
DKTKRO_02	neg	neg	not_expressed	not_expressed	neg	0.020538643
DKTKRO_26	neg	neg	not_expressed	not_expressed	neg	0.020002642
DKTKRO_31	neg	neg	not_expressed	not_expressed	neg	0.018914729
DKTKRO_67	neg	neg	not_expressed	not_expressed	neg	0.018303998
DKTKRO_49	neg	neg	not_expressed	not_expressed	neg	0.01733657
DKTKRO_08	neg	neg	not_expressed	not_expressed	neg	0.01725853
DKTKRO_32	neg	neg	not_expressed	not_expressed	neg	0.014107066
DKTKRO_61	neg	neg	not_expressed	not_expressed	neg	0.013992524
DKTKRO_47	neg	neg	not_expressed	not_expressed	neg	0.013250497
DKTKRO_90	neg	neg	not_expressed	not_expressed	neg	0.013084948
DKTKRO_64	neg	neg	not_expressed	not_expressed	neg	0.011994446
DKTKRO_54	neg	neg	not_expressed	not_expressed	neg	0.011208931
DKTKRO_77	neg	neg	not_expressed	not_expressed	neg	0.010024381
DKTKRO_103	neg	neg	not_expressed	not_expressed	neg	0.009401474
DKTKRO_56	neg	neg	not_expressed	not_expressed	neg	0.009234862

Patient ID	p16/HPV DNA	HPV_E6_E7	HPV16_E6	HPV16_E7	24miRNA predicted status	24miRNA prediction score
DKTKRO_20	neg	neg	not_expressed	not_expressed	neg	0.008967031
DKTKRO_01	neg	NA	NA	NA	neg	0.006171787
DKTKRO_17	neg	neg	not_expressed	not_expressed	neg	0.005975247
DKTKRO_46	neg	neg	not_expressed	not_expressed	neg	0.005184063
DKTKRO_57	neg	neg	not_expressed	not_expressed	neg	0.005141149
DKTKRO_78	neg	neg	not_expressed	not_expressed	neg	0.004702338
DKTKRO_72	neg	neg	not_expressed	not_expressed	neg	0.003866234
DKTKRO_35	neg	neg	not_expressed	not_expressed	neg	0.003494346
DKTKRO_05	neg	neg	not_expressed	not_expressed	neg	0.001617284
DKTKRO_83	neg	neg	not_expressed	not_expressed	neg	0.001540779
DKTKRO_50	neg	neg	not_expressed	not_expressed	neg	0.001127026
DKTKRO_38	neg	neg	not_expressed	not_expressed	neg	0.001118476
DKTKRO_65	neg	neg	not_expressed	not_expressed	neg	0.000684314
DKTKRO_116	neg	neg	not_expressed	not_expressed	neg	0.00063423
DKTKRO_128	neg	neg	not_expressed	not_expressed	neg	0.000553778
DKTKRO_43	neg	neg	not_expressed	not_expressed	neg	0.000524951
DKTKRO_44	neg	neg	not_expressed	not_expressed	neg	0.000434844
DKTKRO_34	neg	neg	not_expressed	not_expressed	neg	7.80383E-05
DKTKRO_74	neg	neg	not_expressed	not_expressed	neg	4.06333E-05

neg: negative, pos: positive; NA: not available

Supplementary Table S4

LMU-KKG cohort – HPV status (p16/HPV DNA, E6/E7 RNA) and 24miRNA prediction

Patient ID	p16/HPV DNA	HPV_E6_E7	HPV16_E6	HPV16_E7	HPV33_E6	HPV33_E7	HPV68_E6	HPV68_E7	24miRNA predicted status	24miRNA prediction score	RNA-seq 15 *vs.* 15
KKG_94	pos	pos	expressed	expressed	not_expressed	not_expressed	not_expressed	not_expressed	pos	0.998828954	yes
KKG_86	pos	pos	expressed	expressed	not_expressed	not_expressed	not_expressed	not_expressed	pos	0.998050453	yes
KKG_43	pos	pos	expressed	expressed	not_expressed	not_expressed	not_expressed	not_expressed	pos	0.99790066	yes
KKG_101	pos	pos	expressed	expressed	not_expressed	not_expressed	not_expressed	not_expressed	pos	0.99742662	yes
KKG_25	neg	neg	not_expressed	not_expressed	not_expressed	not_expressed	not_expressed	not_expressed	pos	0.996010771	no
KKG_81	pos	pos	expressed	expressed	not_expressed	not_expressed	not_expressed	not_expressed	pos	0.992230487	yes
KKG_95	pos	pos	expressed	expressed	not_expressed	not_expressed	not_expressed	not_expressed	pos	0.988573297	yes
KKG_88	pos	pos	expressed	expressed	not_expressed	not_expressed	not_expressed	not_expressed	pos	0.987123868	yes
KKG_85	pos	pos	expressed	expressed	not_expressed	not_expressed	not_expressed	not_expressed	pos	0.970871645	yes
KKG_80	pos	pos	expressed	expressed	not_expressed	not_expressed	not_expressed	not_expressed	pos	0.95048756	yes
KKG_84	neg	NA	NA	NA	NA	NA	NA	NA	pos	0.947512459	no
KKG_96	pos	pos	expressed	expressed	not_expressed	not_expressed	not_expressed	not_expressed	pos	0.924990623	yes
KKG_102	pos	pos	expressed	expressed	not_expressed	not_expressed	not_expressed	not_expressed	pos	0.912188268	yes
KKG_79	neg	NA	NA	NA	NA	NA	NA	NA	pos	0.89441633	no
KKG_99	pos	pos	expressed	expressed	not_expressed	not_expressed	not_expressed	not_expressed	pos	0.829167689	yes
KKG_10	neg	NA	NA	NA	NA	NA	NA	NA	pos	0.812848291	no
KKG_87	pos	NA	NA	NA	NA	NA	NA	NA	pos	0.778897582	no
KKG_93	neg	NA	NA	NA	NA	NA	NA	NA	pos	0.750159014	no
KKG_82	pos	pos	expressed	expressed	not_expressed	not_expressed	not_expressed	not_expressed	pos	0.728717475	yes
KKG_83	pos	NA	NA	NA	NA	NA	NA	NA	pos	0.715560008	no
KKG_98	pos	pos	expressed	expressed	not_expressed	not_expressed	not_expressed	not_expressed	pos	0.679467717	yes
KKG_41	neg	neg	not_expressed	not_expressed	not_expressed	not_expressed	not_expressed	not_expressed	pos	0.652572374	no
KKG_89	pos	pos	expressed	expressed	not_expressed	not_expressed	not_expressed	not_expressed	pos	0.641811068	yes
KKG_60	neg	NA	NA	NA	NA	NA	NA	NA	pos	0.578059265	no
KKG_65	neg	NA	NA	NA	NA	NA	NA	NA	pos	0.549643828	no
KKG_76	neg	NA	NA	NA	NA	NA	NA	NA	neg	0.497614646	no
KKG_56	neg	neg	not_expressed	not_expressed	not_expressed	not_expressed	not_expressed	not_expressed	neg	0.496136096	no
KKG_66	neg	NA	NA	NA	NA	NA	NA	NA	neg	0.495113956	no
KKG_72	neg	neg	not_expressed	not_expressed	not_expressed	not_expressed	not_expressed	not_expressed	neg	0.482380635	no
KKG_27	neg	neg	not_expressed	not_expressed	not_expressed	not_expressed	not_expressed	not_expressed	neg	0.473684106	no
KKG_14	neg	neg	not_expressed	not_expressed	not_expressed	not_expressed	not_expressed	not_expressed	neg	0.471451225	no
KKG_45	neg	NA	NA	NA	NA	NA	NA	NA	neg	0.458074555	no
KKG_16	neg	neg	not_expressed	not_expressed	not_expressed	not_expressed	not_expressed	not_expressed	neg	0.45733692	no
KKG_09	neg	neg	not_expressed	not_expressed	not_expressed	not_expressed	not_expressed	not_expressed	neg	0.453211696	no
KKG_91	pos	pos	expressed	expressed	not_expressed	not_expressed	not_expressed	not_expressed	neg	0.434138644	no
KKG_100	neg	neg	not_expressed	not_expressed	not_expressed	not_expressed	not_expressed	not_expressed	neg	0.343888489	no
KKG_30	neg	neg	not_expressed	not_expressed	not_expressed	not_expressed	not_expressed	not_expressed	neg	0.307785559	no
KKG_08	pos	pos	expressed	expressed	not_expressed	not_expressed	not_expressed	not_expressed	neg	0.300908027	no
KKG_77	neg	neg	not_expressed	not_expressed	not_expressed	not_expressed	not_expressed	not_expressed	neg	0.285907914	no
KKG_64	neg	NA	NA	NA	NA	NA	NA	NA	neg	0.24071225	no
KKG_35	neg	neg	not_expressed	not_expressed	not_expressed	not_expressed	not_expressed	not_expressed	neg	0.225909002	no
KKG_39	neg	neg	not_expressed	not_expressed	not_expressed	not_expressed	not_expressed	not_expressed	neg	0.210254753	no
KKG_02	neg	neg	not_expressed	not_expressed	not_expressed	not_expressed	not_expressed	not_expressed	neg	0.206335833	no
KKG_28	neg	neg	not_expressed	not_expressed	not_expressed	not_expressed	not_expressed	not_expressed	neg	0.182803191	no
KKG_59	neg	neg	not_expressed	not_expressed	not_expressed	not_expressed	not_expressed	not_expressed	neg	0.172184808	no
KKG_75	pos	pos	expressed	expressed	not_expressed	not_expressed	not_expressed	not_expressed	neg	0.154130308	no
KKG_55	neg	neg	not_expressed	not_expressed	not_expressed	not_expressed	not_expressed	not_expressed	neg	0.153640925	no
KKG_97	pos	NA	NA	NA	NA	NA	NA	NA	neg	0.135407008	no
KKG_03	neg	neg	not_expressed	not_expressed	not_expressed	not_expressed	not_expressed	not_expressed	neg	0.132807522	no
KKG_74	neg	NA	NA	NA	NA	NA	NA	NA	neg	0.132765581	no
KKG_58	neg	NA	NA	NA	NA	NA	NA	NA	neg	0.130916432	no
KKG_47	neg	neg	not_expressed	not_expressed	not_expressed	not_expressed	not_expressed	not_expressed	neg	0.121840941	no
KKG_68	neg	neg	not_expressed	not_expressed	not_expressed	not_expressed	not_expressed	not_expressed	neg	0.089095547	no
KKG_12	neg	neg	not_expressed	not_expressed	not_expressed	not_expressed	not_expressed	not_expressed	neg	0.085149775	no
KKG_06	neg	neg	not_expressed	not_expressed	not_expressed	not_expressed	not_expressed	not_expressed	neg	0.084875255	no
KKG_15	neg	neg	not_expressed	not_expressed	not_expressed	not_expressed	not_expressed	not_expressed	neg	0.081273964	no
KKG_67	neg	neg	not_expressed	not_expressed	not_expressed	not_expressed	not_expressed	not_expressed	neg	0.078243887	no
KKG_49	neg	neg	not_expressed	not_expressed	not_expressed	not_expressed	not_expressed	not_expressed	neg	0.060550276	no
KKG_07	neg	NA	NA	NA	NA	NA	NA	NA	neg	0.055585532	no
KKG_29	neg	neg	not_expressed	not_expressed	not_expressed	not_expressed	not_expressed	not_expressed	neg	0.049429339	no
KKG_63	neg	neg	not_expressed	not_expressed	not_expressed	not_expressed	not_expressed	not_expressed	neg	0.043172047	no
KKG_11	neg	neg	not_expressed	not_expressed	not_expressed	not_expressed	not_expressed	not_expressed	neg	0.042604557	no
KKG_57	neg	NA	NA	NA	NA	NA	NA	NA	neg	0.030457999	no
KKG_61	neg	NA	NA	NA	NA	NA	NA	NA	neg	0.029691588	no
KKG_31	neg	neg	not_expressed	not_expressed	not_expressed	not_expressed	not_expressed	not_expressed	neg	0.026478785	no
KKG_32	neg	neg	not_expressed	not_expressed	not_expressed	not_expressed	not_expressed	not_expressed	neg	0.019117256	no
KKG_46	neg	neg	not_expressed	not_expressed	not_expressed	not_expressed	not_expressed	not_expressed	neg	0.015112011	no

Patient ID	p16/HPV DNA	HPV_E6_E7	HPV16_E6	HPV16_E7	HPV33_E6	HPV33_E7	HPV68_E6	HPV68_E7	24miRNA predicted status	24miRNA prediction score	RNA-seq 15 *vs.* 15
KKG_70	neg	neg	not_expressed	not_expressed	not_expressed	not_expressed	not_expressed	not_expressed	neg	0.014463434	no
KKG_05	neg	neg	not_expressed	not_expressed	not_expressed	not_expressed	not_expressed	not_expressed	neg	0.014128968	no
KKG_18	neg	neg	not_expressed	not_expressed	not_expressed	not_expressed	not_expressed	not_expressed	neg	0.014085494	no
KKG_50	neg	neg	not_expressed	not_expressed	not_expressed	not_expressed	not_expressed	not_expressed	neg	0.013714065	no
KKG_40	neg	NA	NA	NA	NA	NA	NA	NA	neg	0.010082555	no
KKG_78	neg	neg	not_expressed	not_expressed	not_expressed	not_expressed	not_expressed	not_expressed	neg	0.0082174	no
KKG_37	neg	NA	NA	NA	NA	NA	NA	NA	neg	0.007950983	no
KKG_69	neg	neg	not_expressed	not_expressed	not_expressed	not_expressed	not_expressed	not_expressed	neg	0.007926187	no
KKG_17	neg	neg	not_expressed	not_expressed	not_expressed	not_expressed	not_expressed	not_expressed	neg	0.007627718	no
KKG_01	neg	NA	NA	NA	NA	NA	NA	NA	neg	0.007527299	no
KKG_51	neg	neg	not_expressed	not_expressed	not_expressed	not_expressed	not_expressed	not_expressed	neg	0.007372273	yes
KKG_22	neg	neg	not_expressed	not_expressed	not_expressed	not_expressed	not_expressed	not_expressed	neg	0.007161392	yes
KKG_34	neg	NA	NA	NA	NA	NA	NA	NA	neg	0.007042113	no
KKG_53	neg	neg	not_expressed	not_expressed	not_expressed	not_expressed	not_expressed	not_expressed	neg	0.006711667	yes
KKG_13	pos	pos	not_expressed	not_expressed	expressed	expressed	not_expressed	not_expressed	neg	0.005543502	no
KKG_90	neg	neg	not_expressed	not_expressed	not_expressed	not_expressed	not_expressed	not_expressed	neg	0.005174718	yes
KKG_73	neg	neg	not_expressed	not_expressed	not_expressed	not_expressed	not_expressed	not_expressed	neg	0.004852906	yes
KKG_92	pos	pos	not_expressed	not_expressed	not_expressed	not_expressed	expressed	expressed	neg	0.004512419	no
KKG_44	neg	neg	not_expressed	not_expressed	not_expressed	not_expressed	not_expressed	not_expressed	neg	0.004128541	no
KKG_38	neg	neg	not_expressed	not_expressed	not_expressed	not_expressed	not_expressed	not_expressed	neg	0.003837574	yes
KKG_71	neg	NA	NA	NA	NA	NA	NA	NA	neg	0.003780474	no
KKG_23	neg	neg	not_expressed	not_expressed	not_expressed	not_expressed	not_expressed	not_expressed	neg	0.003769508	yes
KKG_04	neg	neg	not_expressed	not_expressed	not_expressed	not_expressed	not_expressed	not_expressed	neg	0.003673485	yes
KKG_36	neg	neg	not_expressed	not_expressed	not_expressed	not_expressed	not_expressed	not_expressed	neg	0.003048485	no
KKG_19	neg	NA	NA	NA	NA	NA	NA	NA	neg	0.002545168	no
KKG_54	neg	neg	not_expressed	not_expressed	not_expressed	not_expressed	not_expressed	not_expressed	neg	0.002431508	yes
KKG_52	neg	NA	NA	NA	NA	NA	NA	NA	neg	0.001373513	no
KKG_20	neg	neg	not_expressed	not_expressed	not_expressed	not_expressed	not_expressed	not_expressed	neg	0.000580384	yes
KKG_24	neg	neg	not_expressed	not_expressed	not_expressed	not_expressed	not_expressed	not_expressed	neg	0.000549375	yes
KKG_26	neg	neg	not_expressed	not_expressed	not_expressed	not_expressed	not_expressed	not_expressed	neg	0.000540793	yes
KKG_62	neg	neg	not_expressed	not_expressed	not_expressed	not_expressed	not_expressed	not_expressed	neg	0.000354798	yes
KKG_48	neg	neg	not_expressed	not_expressed	not_expressed	not_expressed	not_expressed	not_expressed	neg	0.000284391	yes
KKG_21	neg	NA	NA	NA	NA	NA	NA	NA	neg	0.000217654	no
KKG_42	neg	neg	not_expressed	not_expressed	not_expressed	not_expressed	not_expressed	not_expressed	neg	8.65448E-05	yes

neg: negative, pos: positive; NA: not available

Supplementary Table S5

Clinicopathological characteristics of HNSCC patient subgroups p16/HPV DNA positive and 24-miRNA signature negative (n = 10) versus p16/HPV DNA negative and 24-miRNA signature positive (n = 11)

	**LMU-KKG and DKTK-ROG**	
**Parameter**	**p16/HPV DNA positive and 24-miRNA signature negative (n = 10)**	**p16/HPV DNA negative and 24-miRNA signature positive (n = 11)**
**Age (years)**		
<45	1 (10%)	0 (0%)
45-54	3 (30%)	4 (36%)
55-64	2 (20%)	4 (36%)
65-74	4 (40%)	3 (27%)
>75	0 (0%)	0 (0%)
**Sex**		
Male	7 (70%)	10 (91%)
Female	3 (30%)	1 (9%)
**Tumour localization**		
Hypopharynx	0 (0%)	5 (45%)
Oral cavity	5 (50%)	1 (9%)
Oropharynx	5 (50%)	5 (45%)
**UICC TNM stage**		
I	0 (0%)	0 (0%)
II	0 (0%)	0 (0%)
III	2 (20%)	1 (9%)
IV	8 (80%)	10 (91%)
**T stage**		
T1	1 (10%)	2 (18%)
T2	2 (10%)	8 (73%)
T3	3 (10%)	0 (0%)
T4	4 (10%)	1 (9%)
**N stage**		
N0	3 (30%)	1 (9%)
N1	0 (0%)	7 (64%)
N2	6 (60%)	1 (9%)
N3	1 (10%)	2 (18%)
**Lymphovascular invasion (LVI)**		
0	6 (60%)	6 (55%)
1	3 (30%)	4 (36%)
Missing information	1 (10%)	1 (9%)
**Venous tumour invasion (VTI)**		
0	8 (80%)	9 (82%)
1	0 (0%)	1 (9%)
Missing information	2 (20%)	1 (9%)
**Perineural invasion (PNI)**		
0	3 (30%)	5 (45%)
1	1 (10%)	0 (0%)
Missing information	6 (60%)	6 (55%)
**Resection margin status**		
0	7 (70%)	9 (82%)
1	3 (30%)	2 (18%)
**ECE**		
Not applicable (N0)	3 (30%)	1 (9%)
No	4 (40%)	4 (36%)
Yes	3 (30%)	6 (55%)
**Smoking status**		
Missing information	3 (30%)	0 (0%)
Nonsmoker	3 (30%)	1 (9%)
Smoker	4 (40%)	10 (91%)
**Simultaneous chemotherapy**		
Yes	8 (80%)	9 (82%)
No	2 (20%)	2 (18%)

Supplementary Table S6

Clinicopathological characteristics of HNSCC patients included in RNA-seq analysis: p16/HPV DNA negative and 24-miRNA signature negative (n = 15) versus p16/HPV DNA positive and 24-miRNA signature positive (n = 15) HNSCC patients.

Parameter	LMU-KKG p16/HPV DNA negative and 24-miRNA negative (n = 15)	LMU-KKG p16/HPV DNA positive and 24-miRNA positive (n = 15)	*p*-value
**Age (years)**			
<45	0 (0%)	1 (4.3%)	
45-54	4 (27%)	2 (13%)	
55-64	7 (47%)	8 (53%)	
65-74	4 (27%)	2 (13%)	
>75	0 (0%)	2 (13%)	
**Sex**			0.439
Male	9 (60%)	11 (73%)	
Female	6 (40%)	4 (27%)	
**Tumour localization**			
Hypopharynx	1 (7%)	1 (4.3%)	
Oral cavity	6 (40%)	0 (0.0%)	
Oropharynx	8 (53%)	14 (93%)	
**UICC TNM stage**			0.827
I	0 (0%)	0 (0%)	
II	1 (7%)	2 (13%)	
III	3 (20%)	3 (20%)	
IV	11 (73%)	10 (67%)	
**T stage**			0.061
T1	1 (7%)	3 (20%)	
T2	4 (27%)	9 (60%)	
T3	9 (60%)	2 (13%)	
T4	1 (7%)	1 (7%)	
**N stage**			0.632
N0	2 (13%)	4 (27%)	
N1	3 (20%)	2 (13%)	
N2	10 (67%)	9 (60%)	
N3	0 (0%)	0 (0%)	
**Lymphovascular invasion (LVI)**			0.686
0	9 (60%)	10 (67%)	
1	5 (33%)	4 (27%)	
Missing information	1 (7%)	1 (7%)	
**Venous tumour invasion (VTI)**			
0	14 (93%)	13 (87%)	
1	1 (7%)	0 (0%)	
Missing information	0 (0%)	2 (13%)	
**Perineural invasion (PNI)**			0.202
0	5 (33%)	9 (60%)	
1	4 (27%)	2 (13%)	
Missing information	6 (40%)	4 (27%)	
**Resection margin status**			0.339
0	10 (67%)	9 (60%)	
1	3 (20%)	6 (40%)	
2	0 (0%)	0 (0%)	
Missing information	2 (13%)	0 (0%)	
**ECE**			0.502
Not applicable (N0)	2 (13%)	4 (27%)	
No	9 (60%)	6 (40%)	
Yes	4 (27%)	5 (33%)	
Missing information	0 (0%)	0 (0%)	
**Smoking status**			0.208
Missing information	1 (7%)	3 (20%)	
Non-smoker	1 (7%)	3 (20%)	
Smoker	13 (87%)	9 (60%)	
**Simultaneous chemotherapy**			
Yes	12 (80%)	11 (73%)	0.081
No	3 (20%)	4 (27%)	

Supplementary Table S7

Differentially expressed genes (n = 658; FDR adjusted *p*-value < 0.1 and |log2 fold change| > 0.5) in 24-miRNA signature-positive/HPV-positive (n = 15) versus 24-miRNA signature-negative/HPV-negative (n = 15) HNSCC.

Gene	log2 fold change	*p*-value	adjusted *p*-value
ABCA13	2.318848715	0.002759711	0.077305173
ABCA17P	4.400914314	3.72735E-11	3.30019E-08
ACP7	-3.132141444	2.96522E-05	0.00306464
ACSS3	-1.641096322	0.000651595	0.029952012
ACTG2	-3.069339802	0.000136119	0.009495939
ACTN1	-1.76134038	1.3764E-07	5.91185E-05
ACTR3C	1.629309521	0.000220257	0.01530247
ADAM12	-1.707966483	0.000934964	0.037566739
ADAMTS12	-1.787349907	0.000537621	0.032988191
ADAMTS15	-3.107323631	3.07001E-07	8.03472E-05
ADGRD1-AS1	-2.98940835	0.00089102	0.035945679
ADGRD2	2.483131478	0.003322524	0.086344137
ADH6	1.988218818	0.00113814	0.040123043
ADPRHL1	-2.524602217	0.000756669	0.032815825
AHNAK2	-1.666423602	0.001746429	0.065005043
AHR	-1.224755917	0.000863797	0.040123043
AJAP1	-3.652214858	2.6415E-06	0.000410632
AKR1C2	-2.476258203	0.000778831	0.040089848
AKR1C3	-2.868501717	4.32409E-05	0.004571024
ALDH1L2	-1.397088147	0.000892756	0.04601446
ALOX12B	-3.231041976	0.000773187	0.030493102
ALOXE3	-4.056265755	7.62637E-06	0.000987959
AMIGO2	-1.925295448	0.000256119	0.016750889
ANO1	-2.478929079	2.19818E-10	9.16956E-07
ANO1-AS1	-2.254364137	0.000576258	0.027141098
APBB2	-1.250600077	0.001227278	0.056402003
APCDD1	-1.444209057	0.000883409	0.044667928
APCDD1L-DT	-2.70393536	0.000263419	0.009435815
APOD	-2.628755142	0.000280309	0.014688242
AREG	-2.525448942	1.99684E-07	5.91185E-05
ARG1	-2.810403303	0.004351236	0.066498666
ARHGAP26	1.861983885	4.07354E-06	0.000917294
ARHGEF25	-1.599377128	0.001982699	0.061448281
ARHGEF33	1.562489769	0.001355356	0.030810631
ARL4AP1	1.638880207	0.001820014	0.056363089
ARMCX1	-1.6551603	0.002954793	0.076705461
ARSI	-2.03983306	0.002762844	0.077213319
ASAP3	-1.32256061	0.000987777	0.043266035
B4GALNT1	-2.261640983	0.000649755	0.032557247
B4GALNT3	-2.077348006	0.000262719	0.025303292
BAALC	-2.114270442	0.000875099	0.035701598
BARD1	1.369755676	3.53707E-05	0.005623442
BCAT1	-1.575777537	0.001510882	0.074359519
BCHE	-4.191899389	9.10484E-05	0.007171791
BCL2	2.048036053	8.08562E-06	0.001413215
BEND6	-2.344301434	3.81005E-05	0.00348907
BGN	-1.77843586	0.000910959	0.045439928
BLID	-3.019612791	0.000732658	0.040967206
BMPR1B	-2.728329485	1.74394E-06	0.000377985
BOC	-1.604992552	0.000857675	0.038686519
BPIFC	-4.747165842	1.6533E-05	0.001969034

Gene	log2 fold change	*p*-value	adjusted *p*-value
BRIP1	1.248904669	0.000735205	0.040123043
BTN2A2	1.369244796	0.002085968	0.07066335
C11orf87	-3.589406762	0.001294361	0.061222665
C17orf51	-1.259083088	0.002787709	0.068058068
C19orf57	2.343773331	5.739E-07	0.000119841
C4orf19	1.867512111	0.002373463	0.06910106
C5orf38	-2.859686166	0.000881686	0.045004395
C5orf46	-4.127901797	8.19131E-07	0.000207308
C5orf56	1.180194292	0.001013381	0.050330372
C5orf66-AS1	2.700442455	0.002213887	0.079576706
C6orf15	-3.30917043	0.002365427	0.09150778
CACNG8	-3.259440875	0.001568853	0.052417656
CALB2	-4.043135044	1.02714E-06	0.000249954
CALD1	-1.514046091	0.00186719	0.068058068
CALHM5	-1.709259957	0.000469523	0.024080908
CAMK2N1	-2.042321603	0.000487137	0.027141098
CAPN14	3.00178105	0.000580941	0.030747971
CARMN	-1.613756116	0.000173425	0.013260627
CASP14	-3.597707047	0.000934184	0.037336087
CAV1	-2.344647496	2.87225E-08	4.89038E-05
CAVIN1	-1.447744101	0.000253746	0.018774853
CAVIN3	-2.295551493	1.68211E-05	0.001182045
CAVIN4	-2.047083145	0.000862637	0.033885302
CCBE1	-2.24141961	0.003487923	0.090121806
CCDC144NL-AS1	-3.849681994	6.12695E-05	0.004571024
CCDC3	-1.889765621	6.07122E-05	0.004555475
CCDC60	2.620094639	0.001929615	0.061222665
CCDC86	-1.434736886	0.000248818	0.015041719
CCNA1	-4.896016738	2.30862E-07	6.45873E-05
CCNE2	1.355898538	0.002421691	0.077412466
CD22	2.856285686	0.000254257	0.016704812
CD248	-1.801763246	0.000875775	0.035945679
CD36	-2.178108681	0.000136363	0.01059078
CD48	1.523036169	0.002087165	0.057149213
CDA	-3.217224954	0.000111364	0.008219087
CDC7	1.281170738	0.000134414	0.010154327
CDH13	-1.983059286	2.51665E-06	0.00050989
CDH19	-3.259465349	8.13798E-05	0.006600814
CDH4	-2.660744116	0.001776579	0.064354313
CDHR1	-2.478274539	4.50486E-05	0.004687742
CDK6	-1.619856436	0.00010431	0.009937295
CDKN2A	2.289792215	0.000251567	0.016628226
CDKN2B-AS1	2.576960365	2.9779E-08	9.83631E-06
CDKN2C	2.010700216	1.19761E-07	3.89489E-05
CDR2L	-1.567612657	0.003100683	0.082615719
CENPK	1.498762233	1.49056E-05	0.002012169
CENPW	1.561290351	0.003153453	0.083337533
CERCAM	-1.769886113	0.00126896	0.048969226
CES1	-3.790011872	2.73844E-05	0.002916528
CFP	1.846276516	0.000814955	0.034526881
CHGB	-3.989593283	5.72574E-06	0.00089678
CIITA	1.260614692	0.003266092	0.086167076
CLEC11A	-2.470199472	0.000225785	0.012535413
CLEC18B	-2.81206006	0.000462748	0.028768389
CLGN	2.814180554	0.002382592	0.044393265
CLMP	-1.612077367	0.001020682	0.044193926
CLTCL1	-1.60719308	0.001177628	0.060543191
CNTN5	-3.265822722	0.000753119	0.032148717
COL12A1	-1.76869924	0.000466396	0.027141098

**Gene**	**log2 fold change**	***p*-value**	**adjusted *p*-value**
COL19A1	2.857618096	0.000994145	0.038214319
COL1A1	-1.897296667	0.001482517	0.065005043
COL1A2	-1.816844751	0.001378854	0.048955818
COL25A1	-3.682120991	0.000166389	0.013779092
COL3A1	-1.854657009	0.001038109	0.040123043
COL4A4	2.012634466	0.000242937	0.01639756
COL4A6	-3.094759235	3.19365E-05	0.007013336
COL5A1	-2.003324642	8.09124E-05	0.008238674
COL5A2	-1.937729759	0.000281284	0.016424164
COL5A3	-1.810805719	0.000133624	0.011676298
COL6A1	-2.282525638	1.01299E-06	0.000252959
COL6A2	-2.363173363	7.02801E-07	0.000205813
COL6A3	-1.718224319	0.000212686	0.017069286
COL6A4P1	2.069299466	0.000817966	0.031308353
CORIN	-1.791625453	0.000302323	0.016770025
CORO6	-2.943713615	3.02602E-06	0.000568527
CPS1	-2.915757709	0.000137376	0.009649897
CPSF1P1	-2.20854109	0.000635023	0.030747971
CR2	3.959371688	0.001314375	0.046771062
CREB3L1	-1.859281274	0.00225441	0.060543191
CREB5	-1.803496463	0.000470205	0.026870065
CREG2	-3.187671987	5.56867E-05	0.004834902
CRIM1	-1.341904939	0.000692463	0.038686519
CSMD3	-4.18337472	0.000860028	0.03559425
CSPG4	-1.926359584	0.000106128	0.010067338
CTTN	-1.152924967	0.001675407	0.067611287
CWH43	-3.498160073	0.001473094	0.050170824
CXCL14	-3.143788214	2.14298E-10	2.92376E-07
CXCL17	2.514745243	4.35958E-06	0.000584844
CXCR2P1	1.902060743	0.00105123	0.040089848
CYCSP6	-3.923378108	0.001705433	0.068058068
CYP26B1	-1.68904805	0.002875471	0.077783207
CYP4F22	-2.885302716	0.001966386	0.061222665
CYP4F30P	-3.684847412	0.003946937	0.098077562
CYP4X1	2.911115838	6.23873E-09	4.00995E-06
CYR61	-1.612474693	0.002332364	0.082633353
DACH1	-2.25539025	0.005209628	0.075115903
DACT1	-2.184189795	0.000613334	0.031224387
DBIL5P2	2.543909483	0.000203152	0.011553971
DCBLD1	-1.345470838	0.000630897	0.036491196
DENND2D	1.094096086	0.001102621	0.05084661
DGKG	-1.594122263	0.001205837	0.040089848
DGKI	-2.043594081	0.000138797	0.010735057
DKK1	-3.853248931	9.21571E-06	0.001182045
DKK3	-1.897107988	7.9441E-05	0.008219087
DLX1	-2.086445537	0.004250106	0.065228347
DLX5	-1.72067982	0.00208844	0.063603641
DMKN	-2.867880396	6.37306E-05	0.008219087
DNAH17-AS1	-2.871529125	0.001159292	0.040620958
DNAJC6	-2.440703765	0.001298917	0.046527074
DNER	-4.047716733	1.37713E-06	0.000275875
DOC2B	1.727281391	0.000137684	0.012572584
DPF1	-2.373202578	0.000948723	0.037184952
DSC1	-6.57285926	1.82566E-11	2.692E-08
DSEL	-1.63628404	0.000352333	0.017807293
DSG1	-5.899817386	5.68794E-12	1.12209E-08
DUOX2	1.817979528	0.002123049	0.078805664
E2F8	1.449648896	0.001883757	0.06587777
EDARADD	1.715852797	0.001021412	0.04386847

Gene	log2 fold change	*p*-value	adjusted *p*-value
EFEMP1	-1.585829448	0.002228849	0.079704194
EFEMP2	-1.443358749	0.000383569	0.028200153
EFNB3	-2.790308823	4.45933E-05	0.004105959
EFR3B	-2.036052671	0.000274773	0.014452304
EGFR	-1.625643531	0.000752243	0.040765099
ELF3	1.814954605	0.000741612	0.045868367
ELFN2	-4.39964213	5.23145E-06	0.000954801
ELOA3	3.347691504	0.00401348	0.097787095
EMC3-AS1	1.337494868	0.000712899	0.031288248
EMILIN1	-1.803568825	0.003590779	0.079349694
EML1	-1.577848806	3.58467E-05	0.0039522
ENPEP	-1.763537912	0.000170368	0.011283534
EPGN	-3.609433547	1.21829E-05	0.00139713
ERBB4	-2.983765655	0.001602274	0.057149213
EREG	-2.978721877	0.001733526	0.062339921
EVA1A	-3.627303287	1.83375E-08	8.37003E-06
EYA2	2.0929699	0.000107554	0.008960683
EZH2	1.196823013	0.001029566	0.044374578
F2R	-1.320029316	0.001252373	0.065228347
F2RL1	-2.36945314	2.90732E-06	0.001158257
FABP4	-3.86801783	6.65543E-05	0.003352763
FAM111B	1.563299729	0.000162241	0.017284613
FAM216B	-2.249576505	0.000541623	0.026713081
FAM221A	1.35534939	0.003366679	0.097293383
FAM222A	1.837894227	0.00468761	0.09967129
FAM25A	-3.190925515	0.003968441	0.097685371
FAM30A	2.083545903	0.000391823	0.026516406
FAM3B	3.286340128	2.12091E-06	0.000391078
FAM81A	2.394668495	0.000215375	0.013260627
FAM83E	2.230852003	0.001278963	0.045811812
FAM89A	-1.648167267	6.25945E-05	0.005290025
FAP	-1.957135588	9.64312E-05	0.009344405
FAR2P4	-5.900514927	1.85944E-07	5.44225E-05
FBN2	-2.134654449	0.000893859	0.052362764
FCRL3	2.064605072	0.003308401	0.086307874
FERMT2	-1.835910462	1.43762E-05	0.001969034
FETUB	-3.447191294	0.001826339	0.060233611
FKBP10	-1.78740231	0.000121895	0.009896004
FKBP9	-1.454938348	9.67039E-08	4.45881E-05
FKBP9P1	-2.677024381	3.26233E-06	0.000551416
FLG2	-3.225817532	0.000764006	0.032883485
FLNC	-2.422901314	0.001716534	0.085971602
FLRT2	-2.147949624	0.000198045	0.021150038
FLRT3	-2.207017809	0.002414568	0.077702345
FN1	-2.170042289	0.000145256	0.010117052
FNDC9	1.671714739	0.001597128	0.034120699
FOXA1	2.235722433	0.000169994	0.012614385
FOXF2	-1.892948427	2.7622E-05	0.002968251
FOXRED2	1.543331539	1.91899E-05	0.002791004
FRMD5	1.50676073	9.83202E-05	0.016095376
FSTL1	-1.501506252	0.000932266	0.045439928
FSTL3	-1.839651815	0.001595553	0.053325825
FTH1P20	-1.677141444	0.000848463	0.022242986
FUT6	3.320609893	0.000189827	0.010563841
GABRQ	2.487190516	0.000895794	0.03604725
GALNT13	-3.906785846	1.62408E-07	3.08235E-05
GALNT6	-2.481646745	1.84266E-09	1.36884E-06
GAP43	-3.316784312	0.000548529	0.032815825
GAPDHP54	2.602825916	0.000352893	0.021687605

Gene	log2 fold change	*p*-value	adjusted *p*-value
GATA4	-4.700461369	0.000397679	0.021150038
GBX1	4.299633731	0.000138681	0.010877455
GDF15	2.902693772	0.002939325	0.078367308
GDPD2	-3.573609769	0.001874767	0.0608415
GDPGP1	1.152389393	0.002281524	0.067748085
GHR	-1.684777048	0.001987557	0.062623169
GINS2	1.670142388	4.26484E-05	0.004048936
GJA1	-2.097930432	6.73399E-06	0.00101119
GJB2	-2.323243473	0.000351608	0.024466216
GJB6	-3.08634875	8.18807E-05	0.008219087
GKAP1	1.80114032	0.002385519	0.069290623
GLI2	-2.649655274	5.68714E-05	0.004316054
GOLGA2P5	1.391451096	0.003042969	0.090633178
GPSM1	-1.433048787	0.004319488	0.094427136
GPX2	-2.24833116	0.000812012	0.040967206
GREM1	-2.026908712	2.4756E-05	0.003332174
GSDMA	-3.579310772	0.000187225	0.012151993
HELLS	1.488146539	1.29121E-05	0.001601066
HEPH	-1.55779871	0.001733681	0.057528502
HLF	1.860414467	0.003184112	0.093942359
HMCN1	-1.522695424	0.001750588	0.06910106
HMGB3P8	-4.355471502	4.81137E-05	0.005381016
HMSD	3.71657813	1.40238E-08	6.78903E-06
HOXC13	-2.965375519	2.32193E-07	6.45873E-05
HOXC13-AS	-3.246175185	0.000636621	0.038056025
HS3ST4	5.305824488	8.44423E-07	0.000190305
HSPB3	-3.070981121	0.001488916	0.063938456
HTRA1	-1.83196013	0.000306764	0.022659441
HVCN1	1.687472475	0.002493303	0.068288198
IDO1	2.726856136	4.99121E-06	0.001182045
IGDCC4	-1.797304117	8.34631E-05	0.006600814
IGF1	-1.775242928	0.003327506	0.097467154
IGF2BP1	-6.31344454	1.22513E-07	5.91185E-05
IGFBP6	-3.079733131	9.63055E-09	4.963E-06
IGFL2	-4.778530307	7.03019E-07	0.000152424
IGFL3	-3.279533636	0.001949887	0.078662366
IGHV1-69D	2.748047907	0.001433637	0.06792547
IGHV4-4	2.460425023	0.000331133	0.019006657
IGKV1-33	3.003740351	0.001126256	0.046771062
IGLV1-44	2.505073587	0.003009736	0.080456722
IGLV3-19	2.527256327	0.002250985	0.067216079
IGLV9-49	3.94697893	0.001967688	0.061222665
IKZF2	1.409082098	0.000700091	0.031308353
IKZF3	1.643529615	5.1924E-05	0.005740972
IL1R1	-1.342202061	0.000386973	0.026348854
IL2RG	1.436398967	0.001356769	0.053130072
INHBA	-2.314499885	1.75978E-05	0.002655683
INHBA-AS1	-2.61508606	0.001156167	0.043326674
INHBB	-3.377751465	2.33215E-09	1.49029E-06
INSYN1	-2.098853538	0.003858218	0.095175587
IRS1	-1.489493384	0.001701947	0.070677887
IRX1	-3.573533805	0.001573061	0.079326106
IRX2	-2.582657711	1.12398E-06	0.000261885
ITGA5	-1.718456142	2.58547E-05	0.004141029
ITPRID2	-1.187958251	0.000591548	0.03559425
JAKMIP3	-2.683824004	0.000573803	0.027141098
JPH2	-3.464662857	3.75837E-05	0.003629354
KANK4	-3.0431336	7.27037E-05	0.005958183
KCND2	-2.085801526	0.002679586	0.068662491

Gene	log2 fold change	*p*-value	adjusted *p*-value
KCNIP4-IT1	-4.613115892	3.00753E-05	0.00306464
KCNJ10	1.84319273	0.001407478	0.048880788
KCNK10	-2.844270925	0.003649555	0.060413588
KCNMB4	-2.103853274	0.00058038	0.027141098
KCNS1	2.394700073	0.000261499	0.01530247
KIF3C	-1.556990178	0.001496679	0.057285006
KIRREL1	-1.690890173	8.55745E-07	0.000240839
KLC3	-1.930833675	0.003503628	0.090383672
KLK14	-5.399329254	6.4747E-06	0.000958928
KLK5	-6.531956423	1.41353E-09	1.18965E-06
KLK6	-4.255045589	5.99441E-05	0.004210383
KLK7	-3.796079195	0.000130252	0.010312589
KLK8	-3.357338504	1.10531E-05	0.001411676
KRT1	-5.82021521	1.36586E-08	8.42176E-06
KRT14	-3.110870146	2.09911E-05	0.00308143
KRT15	2.991387168	1.50884E-11	2.692E-08
KRT16	-2.299552874	0.000312643	0.022242986
KRT16P2	-3.478852187	2.99137E-07	7.44193E-05
KRT17	-1.988661131	8.46583E-07	0.000240839
KRT6B	-3.050260469	1.7113E-05	0.002540076
KRT6C	-2.814665624	0.001879328	0.076071528
KRT75	-5.975093851	1.50836E-10	1.73576E-07
KRT79	-3.841204244	0.004615178	0.098430383
KRTDAP	-5.909316115	9.6021E-10	9.16956E-07
KYNU	1.655702658	0.002534582	0.092577761
L1CAM	-3.577885457	7.54675E-07	0.000172233
LAMA4	-1.434700482	0.000466719	0.030493102
LAMC3	-2.142099224	0.002342665	0.077199065
LCE3D	-3.450925431	0.001946282	0.061222665
LCE3E	-4.108564795	0.000109183	0.008642106
LGALS1	-1.49899845	0.001729541	0.06954891
LGALSL	-1.871067776	6.92607E-05	0.009435815
LINC00494	2.849798437	0.00158186	0.065005043
LINC00504	-2.502823393	6.3949E-05	0.005376494
LINC00702	-2.169482142	0.002336552	0.068058068
LINC00707	-3.340526918	1.83724E-05	0.002078312
LINC00885	2.707574711	1.47141E-05	0.001571477
LINC01006	1.598071689	0.001726971	0.057423254
LINC01108	2.248777778	0.003407525	0.086344137
LINC01215	2.231524989	0.002716234	0.083561518
LINC01234	-4.42576643	1.91961E-05	0.002174947
LINC01305	3.532069007	2.91957E-08	1.22741E-05
LINC01429	-2.299941824	0.00239385	0.070068142
LINC01524	-3.606373658	0.000436324	0.027519921
LINC01561	-2.643013201	0.003042839	0.09150778
LINC01614	-2.743637344	0.000980867	0.038126106
LINC01929	-4.74177896	3.16647E-07	8.15564E-05
LINC02614	-1.526910094	2.4697E-05	0.003034561
LIPE-AS1	2.090585197	0.000584338	0.027456147
LIPH	2.344246885	0.00114412	0.046527074
LIPK	-4.176329632	0.000265485	0.019010538
LMO4	1.490596391	0.000514494	0.030970665
LMX1B	-4.192503832	2.03575E-07	6.006E-05
LNCAROD	-4.169947204	2.82695E-07	0.000104722
LOXL2	-2.16358119	5.24251E-06	0.001048371
LRCH2	-1.898298728	0.000905609	0.036197418
LRRC17	-2.080892584	0.003193334	0.08322201
LUZP2	-3.44758118	0.000263617	0.009435815
LY6G6C	-3.862260274	0.000108139	0.008219087

Gene	log2 fold change	*p*-value	adjusted *p*-value
MAATS1	2.810269957	0.002563487	0.072999945
MACC1	1.823533027	0.000123411	0.010960642
MAGEA11	-5.46296121	1.01477E-06	0.000194487
MAGEA4-AS1	-4.459993741	0.00013078	0.012527675
MAGEB2	-5.890467464	0.002315012	0.06276941
MAGI2	-1.34879939	0.000630775	0.029160143
MAGI2-AS3	-1.411937206	0.002520565	0.077213319
MAJIN	3.196209635	0.000233516	0.022573223
MAP1B	-1.611327103	8.81086E-06	0.001480137
MAP2	-2.069721757	1.19059E-05	0.001668595
MAP7D2	3.182483156	2.13829E-06	0.000391078
MB	-2.919783894	0.002200232	0.059513861
MCM5	1.270322851	5.98612E-06	0.001158246
MEDAG	-1.877100244	0.004034983	0.098413508
MEG8	-1.662909369	0.00371007	0.092577761
MEI1	4.637801557	1.45387E-20	1.61359E-16
MEIS1	1.31075415	2.92982E-05	0.003917697
MEIS3	-2.004290038	0.000861193	0.035517541
MFAP2	-1.73914281	0.000318453	0.025303292
MFAP3L	-1.762155354	0.000622445	0.031442431
MFSD4A	1.647357526	1.09695E-05	0.001413215
MICAL2	-1.613042981	0.000334156	0.024372283
MIR9-3HG	3.159001183	9.22507E-08	3.86116E-05
MLXIPL	-3.117260019	0.00028769	0.016424164
MME	-2.197009775	4.95703E-05	0.004989255
MMP13	-5.105382981	1.35032E-11	2.692E-08
MMP16	-2.461185518	7.00883E-06	0.001163355
MMP7	-2.585610402	0.000172613	0.016095376
MN1	-1.763035157	0.001083383	0.045861295
MS4A1	2.814885491	0.001976987	0.075560336
MSRB3	-2.070336788	2.60872E-05	0.003034561
MSX2	-2.699251861	0.000279195	0.017843321
MTMR8	1.841573732	0.000547438	0.026513113
MTND2P28	-2.09427989	0.001244261	0.045074103
MUC4	3.699248712	2.03003E-06	0.000495177
MUCL1	-5.246807679	6.45883E-07	0.000150425
MYB	1.688474762	0.001600553	0.053140474
MYBPC2	-2.768788881	0.001036726	0.040089848
MYF6	-3.756161835	0.000226291	0.013779092
MYH10	-1.424503347	0.000390806	0.026139022
MYH8	-3.240193446	0.003024246	0.080711647
MYLK	-1.265688501	0.002718502	0.094427136
MYO5A	-1.119267646	0.002791993	0.096383318
MYO5C	1.801210644	0.000259603	0.018774853
MYOM3	-2.927749997	4.94282E-05	0.004989255
N4BP3	1.856429077	2.1748E-06	0.000347994
NACAD	-2.199340362	0.000819246	0.035945679
NALCN	-2.79258729	0.000355963	0.018892638
NAP1L2	-2.923608154	5.7938E-08	2.18468E-05
NAV3	-2.061278521	2.58632E-05	0.002385346
NBEAP1	-3.496844126	0.000978806	0.038363573
NCAM1	-2.142493102	0.000731379	0.029160143
NCEH1	1.167500302	0.000463942	0.029256331
NDN	-1.988204651	0.000926551	0.036821179
NEFH	4.330486679	3.00952E-14	9.76675E-11
NELL2	-2.150841735	3.96893E-05	0.004725796
NEXMIF	3.380479562	1.53901E-08	7.16864E-06
NFATC4	-1.329107086	0.003264485	0.094358519
NID1	-2.166452851	4.26158E-07	0.000399482

**Gene**	**log2 fold change**	***p*-value**	**adjusted *p*-value**
NKAIN2	-2.799544838	5.19985E-06	0.0007203
NKD1	-1.851342588	0.000192406	0.01530247
NLRP10	-3.908861275	0.000573707	0.040652168
NMRAL2P	-4.019028864	5.54463E-06	0.001415568
NOS1	-3.741113183	0.000100149	0.006600814
NOS2	5.05801764	1.3186E-07	9.13252E-05
NPR2	-1.83010019	0.000284991	0.01639756
NRG1	-2.009131961	9.42977E-05	0.009344405
NRTN	2.675622028	0.00203498	0.062881661
NSD2	1.019047119	0.001842076	0.060543191
NT5E	-1.507382324	0.001127176	0.046017298
NTM	-1.838296276	0.000965445	0.036197418
NTS	4.317315054	0.000425686	0.035701598
NUP62CL	1.582757669	0.001671456	0.050182634
NWD1	2.448862538	0.002397042	0.069498959
OBSCN	-1.468704512	0.001954854	0.076071528
OLFM2	-2.502211404	4.2743E-07	8.86984E-05
OLFM4	-2.720154221	0.003925878	0.096543882
OLMALINC	1.813803156	0.000165833	0.010902336
OPCML	-3.410158014	0.002140076	0.079576706
OR2B2	2.561955162	0.002910676	0.082633353
OR7E13P	-2.187878638	0.001670313	0.056347592
OR7E14P	2.351415539	0.002763923	0.077213319
OR7E2P	-2.019314876	0.002775508	0.076578067
P3H3	-1.712048688	0.000883352	0.035701598
PALM2	-1.54092964	0.001254757	0.045351509
PAPPA2	-3.125804608	0.000308635	0.017269126
PAQR5	-1.806504857	0.000381557	0.017295077
PART1	2.015155554	0.001512534	0.046611575
PAX1	4.34711794	0.001903527	0.061222665
PAX3	-3.569019622	0.000579946	0.027502561
PAX9	1.926316672	8.89833E-05	0.008960683
PCA3	-3.187582193	0.000944585	0.037566739
PCBD2	1.091524194	0.000529115	0.027502561
PCDH9	-2.170977701	0.001811751	0.064998061
PCDHB4	-3.128947663	9.72474E-05	0.00740438
PCDHGA11	-1.561564557	0.001696315	0.055967911
PCDHGA12	-1.782014974	0.001472855	0.04589425
PCDHGA2	-2.545170988	2.77743E-05	0.001679001
PCDHGA4	-2.870468058	2.85828E-07	7.48565E-05
PCDHGB1	-2.520478403	0.001946574	0.061222665
PCDHGB4	-2.302410412	0.000206583	0.010563841
PCDHGB5	-1.890322545	0.000411984	0.021438607
PCDHGB6	-1.436262443	0.002270362	0.057149213
PCDHGB7	-1.49951901	0.002469393	0.070677887
PCDHGB9P	-1.406419558	0.00245057	0.06786492
PCDHGC3	-1.610790676	0.000739608	0.035701598
PCDHGC4	-1.949449093	0.000236079	0.011676298
PCSK5	-1.899942523	4.96544E-05	0.00740438
PDGFC	-1.613970484	0.00084526	0.038363573
PDPN	-1.530013317	0.002190095	0.094671528
PHACTR2-AS1	1.419485125	0.003396048	0.086329579
PI15	-1.876978462	0.001813402	0.065005043
PI3	-3.102231597	0.000857857	0.050790647
PIK3R3	1.518452704	1.28509E-07	6.35859E-05
PITX1	1.731527489	7.81415E-06	0.001221886
PKIA-AS1	2.206122346	0.003221944	0.083558677
PLA2G16	-2.088139018	6.13959E-05	0.005135236
PLA2G2D	3.056439156	0.000646991	0.027141098

Gene	log2 fold change	*p*-value	adjusted *p*-value
PLAC8	3.873834385	2.76615E-13	9.58077E-10
PLCB4	-1.998395249	0.000723793	0.026805535
PLEKHG4B	-2.533765339	0.000237741	0.020008649
PLK2	-1.375737694	0.00031093	0.021735695
PLPP4	-2.607246979	0.00092378	0.033922508
PLSCR3	-1.27076812	0.001589791	0.060543191
PLXDC2	-1.381543387	0.000639015	0.035690478
PNLIPRP3	-6.81861916	3.0794E-11	3.30019E-08
PODXL2	1.794192191	0.002971579	0.079699308
POPDC3	-2.752033612	0.000338816	0.018448904
POSTN	-2.413318	3.82695E-05	0.004834902
POTEE	-3.577414023	1.23746E-07	4.87824E-05
PRDM15	1.085296116	8.81706E-06	0.001980788
PRDX3P4	-1.924624217	0.002009211	0.062569467
PRICKLE2-AS3	-2.361684597	0.003166013	0.053940975
PRIMA1	1.842281758	0.00400285	0.098077562
PRMT8	-4.529325884	0.002655292	0.09806977
PROSER2-AS1	1.731441023	0.003222509	0.077305173
PRSS12	1.696083892	1.04165E-05	0.001679001
PRSS23	-1.644529877	0.000531982	0.032815825
PSG3	3.350614899	0.003318175	0.085032425
PSG8	3.339625857	0.003016333	0.074692225
PSORS1C1	-2.206947549	0.002047344	0.068914295
PTHLH	-3.117854842	1.67953E-05	0.003034561
PTK7	-1.296623659	0.000421218	0.031544244
PTPRD	-2.489143612	7.71738E-06	0.001204187
PTPRN	-3.689223573	0.000213628	0.013260627
PXDN	-1.859024798	7.61439E-05	0.008048485
PYGL	-1.814296555	0.001485934	0.062623169
QRSL1P1	-4.356803263	4.95975E-09	2.47825E-06
RAB3B	-3.044775245	3.08712E-06	0.000525076
RAD9B	2.216870008	6.19746E-06	0.000532392
RANBP17	2.527563607	1.36693E-07	5.17308E-05
RASSF6	1.68533664	0.002486731	0.078805664
RASSF9	2.099845909	0.000694995	0.049053394
RDH12	-3.663551844	0.000343631	0.01675155
RFPL1S	3.903665979	3.57057E-09	2.04989E-06
RFX8	-2.571412051	0.000303458	0.017807293
RHBDL3	2.418076531	0.000607282	0.028008327
RHOB	-1.589990372	9.0697E-05	0.009068564
RHOBTB1	-1.300242084	0.003003542	0.077213319
RHOD	-2.027069561	0.000478989	0.021687605
RIBC2	2.331779442	2.09793E-05	0.002380553
RMI2	1.381931971	0.001223581	0.049153695
RN7SL471P	-3.952957479	0.002048751	0.070068142
RNF165	2.162465498	0.000526777	0.039163722
RNF212	2.861032825	2.07363E-05	0.004989255
RNF217-AS1	-1.752625973	0.001892065	0.060574507
RPE65	-5.463590271	0.000252837	0.015194966
RPLP0P2	-2.151592359	0.000118547	0.005091888
RPSAP52	-2.690946525	0.002121093	0.065005043
RTKN2	1.416717871	0.000272985	0.026870065
RTL1	-2.217975244	0.002608883	0.077305173
SALL1	-2.754357531	0.000233421	0.015041719
SBSN	-3.321994568	0.002045122	0.077412466
SCARA5	-3.669351132	2.69151E-08	6.78903E-06
SCARF2	-2.068863676	0.001539858	0.052602904
SCG2	-2.905764926	0.000189954	0.013779092
SCIN	2.161900837	0.003953042	0.09807907

Gene	log2 fold change	*p*-value	adjusted *p*-value
SCN9A	-2.310120201	0.000292373	0.016704812
SERPINE1	-2.194218127	2.22911E-05	0.003251689
SERPINI2	4.363164662	1.13103E-05	0.001413215
SFRP2	-2.018120691	0.002110936	0.078662366
SGCD	-1.856280999	0.0014853	0.077783207
SH3RF3	-1.728886814	6.01909E-05	0.004867383
SHANK1	-3.28229112	1.95751E-05	0.002236859
SHANK2	-2.589152772	0.001686978	0.061222665
SHCBP1L	3.985885762	0.000482311	0.031442431
SIM2	2.262395946	0.000227981	0.017295077
SIRPG	1.640776734	0.002891438	0.077744979
SIX6	2.640074154	0.002057971	0.077943181
SLC22A3	-2.839487331	4.36639E-06	0.001015587
SLC24A2	-2.106471966	0.000470446	0.024080908
SLC24A3	-1.833993761	0.000798681	0.03694107
SLC25A21	2.040439125	0.002256532	0.067256016
SLC26A9	2.067077367	0.000525213	0.026222515
SLC29A4	-1.866469181	0.00389685	0.097685371
SLC47A2	-2.327815194	7.56719E-05	0.007013336
SLC4A3	-1.676129085	0.001786538	0.058246599
SLC6A10P	-4.571362522	0.000109916	0.008219087
SLC6A2	-3.32971249	8.48813E-05	0.006366872
SLC7A11	-2.520768467	0.000143324	0.012572584
SLC7A11-AS1	-2.414417028	0.002831733	0.077213319
SLC7A8	-2.009670301	3.1398E-06	0.000958928
SLCO1A2	-3.369901394	0.002355988	0.061352743
SLCO1B3	-4.748056651	0.000121579	0.007725782
SLCO1B7	-4.505713539	0.000894767	0.047660958
SLCO1C1	-2.90827834	2.46027E-05	0.002655683
SLFN13	2.56914075	7.18485E-16	3.89252E-12
SLITRK6	-3.066948144	3.27867E-05	0.003325068
SMC1B	4.259431048	4.61709E-26	2.07855E-21
SMOX	-2.12576504	8.98041E-06	0.001205601
SMTN	-1.319564112	0.001327658	0.076071528
SNAI2	-1.577257448	1.28985E-06	0.000340877
SNAP25-AS1	-3.659282029	0.002493244	0.071126435
SNHG26	-1.419262507	0.001408027	0.097685371
SOHLH2	-3.802524533	0.000470109	0.031224387
SORCS2	-2.007268641	0.000605818	0.023504759
SOSTDC1	-4.334794869	7.57412E-06	0.000618122
SOWAHB	1.658487107	0.002700668	0.075958636
SOX30	2.839486546	4.28321E-05	0.004037699
SPARC	-1.727709109	0.002687767	0.092015505
SPATA12	1.982361315	1.11392E-07	3.09403E-05
SPEG	-2.25529072	0.000119239	0.009649897
SPIB	2.075198327	0.003824621	0.086167076
SPOCD1	-1.954254546	0.003774789	0.094427136
SPOCK1	-2.766645144	2.89235E-06	0.000559512
SPON1	-1.533676225	0.001087634	0.051257854
SPRR2G	-5.178757697	3.08955E-06	0.000525076
SPRR4	-3.521238798	0.000850221	0.035648686
SRCIN1	-1.932103468	0.001542113	0.047211154
SRPX	-3.074172717	2.59786E-07	7.14473E-05
ST6GALNAC1	2.187654867	0.003500382	0.088555361
STAG3	1.65370683	0.000172926	0.01675155
STIL	1.128305851	0.000678459	0.037184952
SULF2	-1.766558264	7.09802E-05	0.006897844
SVIP	1.587038115	0.002204781	0.063583896
SYCP2	3.526050671	1.43979E-16	3.89252E-12

Gene	log2 fold change	*p*-value	adjusted *p*-value
SYCP3	1.859252662	0.001289814	0.063583896
SYNGR3	2.22532891	0.000186746	0.011877507
TAF7L	3.266721068	2.30594E-06	0.000377985
TCAIM	1.036170355	0.002750164	0.083353696
TCAM1P	3.084259739	7.32169E-07	0.000190305
TCF19	1.378542077	0.000351884	0.03064381
TCHH	-3.869725518	4.30975E-08	1.54608E-05
TCHHL1	-3.280271855	0.000605488	0.027794579
TCP11	2.958254421	0.000215586	0.01350649
TDRD10	3.203335711	0.00015064	0.009367018
TDRD12	-4.303785815	0.001946237	0.061222665
TDRD9	3.257697034	0.000281409	0.014103396
TENM2	-3.791032183	3.27967E-10	1.18965E-06
TEX30	1.353804723	0.005338344	0.09930371
TEX41	-2.199769858	0.000366356	0.016222545
TFPI2	-3.347003536	1.24981E-05	0.001522559
TGFBI	-1.918055033	0.000795772	0.04282384
THBS1	-1.508682048	0.000185072	0.013882121
THBS2	-2.211659177	3.42477E-05	0.004513288
TIMP3	-1.801508806	0.00149836	0.051959065
TJP3	2.789286342	1.89609E-06	0.00036627
TLL1	-2.02585617	0.002258038	0.060543191
TLN2	-1.623022113	3.62725E-05	0.004048936
TLR10	2.391850646	3.87671E-05	0.003812434
TMEM158	-2.080517878	0.00056552	0.016424164
TMEM45A	-2.156205976	3.11846E-05	0.005091888
TMEM51-AS1	1.815574116	0.002042194	0.063603641
TMPRSS2	2.698646436	9.53344E-05	0.008219087
TNC	-1.943678747	2.92681E-05	0.00306464
TNFAIP8	1.026458574	0.000500159	0.031442431
TNFRSF13B	2.708678201	0.00046585	0.025235159
TNFRSF13C	1.606175898	0.002301713	0.076071528
TNIP3	2.249255178	0.002820791	0.077213319
TNNT1	-2.534787495	0.001783024	0.047579083
TPM1	-1.624266176	0.000523098	0.031228364
TPM1-AS	-1.61604278	0.003457465	0.0894773
TPM2	-1.65167983	0.002308543	0.076071528
TRABD2B	-2.521376553	1.2435E-05	0.000958928
TRAM1L1	-3.306410199	0.000404215	0.028008327
TRIM73	1.599869541	0.001279506	0.038126106
TSPAN18	-1.61675442	0.000691965	0.027141098
TTC6	3.960295388	6.78064E-13	1.05408E-09
TUBA1A	-1.39468969	0.000504851	0.027502561
TWIST1	-1.888766492	0.000554661	0.025632554
UBTD1	-1.802219876	0.000624103	0.017324097
UNC5C	-2.906210957	1.38744E-09	1.18965E-06
UPB1	3.756219579	5.63329E-08	2.17597E-05
USP2	-1.993370273	0.001601006	0.05084661
VCAM1	2.19756125	0.001895196	0.074588816
VCAN	-1.653446538	0.002123813	0.077783207
VEGFC	-2.530755407	2.26371E-05	0.002494058
VGLL3	-1.70557936	0.000442171	0.032815825
VN1R54P	-4.775355956	0.001061339	0.045439928
WDR49	3.118542495	0.00079241	0.033111947
WDR66	-1.526458832	0.001871642	0.063924837
WDR76	1.478437054	5.59633E-06	0.001410559
WFDC12	-4.239492053	6.18981E-05	0.005958183
WNK3	2.540618582	3.53697E-10	3.61796E-07
XCL1	-3.948608838	3.76083E-09	2.22523E-06

**Gene**	**log2 fold change**	***p*-value**	**adjusted *p*-value**
YBX2	2.025119582	0.00103949	0.044374578
ZBTB8B	4.199467565	0.002608937	0.07351925
ZFPM2	-1.771937582	0.002953118	0.079576706
ZFPM2-AS1	-3.715861666	7.73187E-09	4.08166E-06
ZFR2	5.464623871	2.19871E-13	5.12698E-10
ZMAT1	2.123352782	3.71492E-08	1.73114E-05
ZNF100	1.502793522	0.000765184	0.036197418
ZNF214	1.699528126	0.002494719	0.070677887
ZNF365	-2.196348627	9.46093E-06	0.001249116
ZNF469	-2.303341632	2.57803E-05	0.004680416
ZNF521	-1.467244896	0.002651715	0.078925501
ZNF541	4.382603949	2.3272E-13	5.26514E-10
ZNF718	1.394676605	0.00019515	0.013987354
ZNF969P	-2.87403225	0.002031742	0.069737726
ZPBP2	3.645587779	0.000578706	0.035945679
ZSCAN31	-1.466627899	0.002713098	0.082633353
ZYG11A	1.829225531	3.71446E-05	0.004048936

Supplementary Table S8

Genes likely to be regulated by the signature miRNAs and belonging to the signalling pathways that showed differential activity between HPV-positive and HPV-negative cases in Progeny analysis (*MAP kinase, Estrogen, EGFR, TGFbeta* and *WNT* signalling pathway). Genes were identified by matching the identified statistically significant miRNA-gene interactions with pathway gene sets from the Reactome database.

EGFR signalling pathway

gene	miRNA
ARHGEF7	hsa-miR-181a-5p, hsa-miR-205-3p
CBL	hsa-miR-7-5p, hsa-miR-7851-3p
CDC42	hsa-miR-92b-3p, hsa-miR-18a-5p
EGFR	hsa-miR-7-5p
EPS15	hsa-miR-152-3p, hsa-miR-3174
GAB1	hsa-miR-7-5p
GRB2	hsa-miR-96-5p, hsa-miR-378a-3p
HBEGF	hsa-miR-96-5p
KRAS	hsa-miR-96-5p, hsa-miR-501-3p, hsa-miR-7851-3p, hsa-miR-155-5p
NRAS	hsa-miR-3135b, hsa-miR-152-3p
PAG1	hsa-miR-152-3p, hsa-miR-6763-5p
PIK3CA	hsa-miR-92b-3p, hsa-miR-155-5p
PIK3R1	hsa-miR-96-5p, hsa-miR-455-3p
PLCG1	hsa-miR-96-5p
PTPRK	hsa-miR-92b-3p
PXN	hsa-miR-3135b
SH3GL2	hsa-miR-1234-3p
SH3GL3	hsa-miR-205-3p
SH3KBP1	hsa-miR-96-5p
SHC1	hsa-miR-96-5p, hsa-miR-6763-5p
SOS1	hsa-miR-152-3p, hsa-miR-205-3p, hsa-miR-155-5p
STAM2	hsa-miR-378a-3p
TGFA	hsa-miR-152-3p, hsa-miR-7-5p

Estrogen signalling pathway

gene	miRNA
AGO2	hsa-miR-654-5p
AGO4	hsa-miR-181a-5p, hsa-miR-152-3p, hsa-miR-96-5p, hsa-miR-455-3p
AKT1	hsa-miR-501-3p
AKT3	hsa-miR-654-5p, hsa-miR-181a-5p
CALM1	hsa-miR-181a-5p
CARM1	hsa-miR-654-5p, hsa-miR-181a-5p
CAV1	hsa-miR-205-3p, hsa-miR-96-5p
CHD1	hsa-miR-152-3p
CXCL12	hsa-miR-1238-3p
CXXC5	hsa-miR-92b-3p
EBAG9	hsa-miR-501-3p
EGFR	hsa-miR-7-5p
ELK1	hsa-miR-6763-5p, hsa-miR-3135b
EP300	hsa-miR-96-5p
ESR1	hsa-miR-181a-5p, hsa-miR-152-3p, hsa-miR-18a-5p
FOS	hsa-miR-155-5p, hsa-miR-181a-5p
FOXO3	hsa-miR-96-5p
GNAI1	hsa-miR-181a-5p
GNAI2	hsa-miR-663b, hsa-miR-3135b
GNB2	hsa-miR-7851-3p
GNB3	hsa-miR-6763-5p
GNB4	hsa-miR-96-5p

GNB5	hsa-miR-152-3p, hsa-miR-455-3p
GNG12	hsa-miR-96-5p
GNG2	hsa-miR-181a-5p, hsa-miR-3132, hsa-miR-18a-5p
GNG3	hsa-miR-654-5p
GNG7	hsa-miR-492, hsa-miR-6763-5p
GTF2A1	hsa-miR-92b-3p, hsa-miR-7-5p
HBEGF	hsa-miR-96-5p
IGF1R	hsa-miR-378a-3p, hsa-miR-96-5p, hsa-miR-492
JUN	hsa-miR-92b-3p
KANK1	hsa-miR-181a-5p
KAT2B	hsa-miR-181a-5p, hsa-miR-92b-3p
KRAS	hsa-miR-96-5p, hsa-miR-501-3p, hsa-miR-7851-3p, hsa-miR-155-5p
MAPK1	hsa-miR-378a-3p
MED1	hsa-miR-96-5p
MMP3	hsa-miR-18a-5p
MMP7	hsa-miR-181a-5p
MYB	hsa-miR-96-5p
NCOA1	hsa-miR-18a-5p, hsa-miR-92b-3p
NRAS	hsa-miR-3135b, hsa-miR-152-3p
PIK3CA	hsa-miR-92b-3p, hsa-miR-155-5p
PIK3R1	hsa-miR-96-5p, hsa-miR-455-3p
PIK3R3	hsa-miR-181a-5p
POLR2E	hsa-miR-663b, hsa-miR-6746-5p
POLR2F	hsa-miR-205-3p
POU2F1	hsa-miR-96-5p, hsa-miR-3135b, hsa-miR-654-5p, hsa-miR-18a-5p, hsa-miR-7-5p
PRKCZ	hsa-miR-152-3p
PTGES3	hsa-miR-152-3p, hsa-miR-378a-3p
PTK2	hsa-miR-7-5p
RAD21	hsa-miR-205-3p, hsa-miR-501-3p, hsa-miR-92b-3p, hsa-miR-181a-5p
SHC1	hsa-miR-96-5p, hsa-miR-6763-5p
SMC1A	hsa-miR-455-3p
SP1	hsa-miR-205-3p, hsa-miR-7-5p
SRF	hsa-miR-3135b, hsa-miR-7-5p
STAG2	hsa-miR-181a-5p, hsa-miR-92b-3p
TBP	hsa-miR-152-3p, hsa-miR-654-5p
TFF3	hsa-miR-7-5p
TGFA	hsa-miR-152-3p, hsa-miR-7-5p
TNRC6A	hsa-miR-152-3p, hsa-miR-654-5p, hsa-miR-96-5p, hsa-miR-7-5p
TNRC6B	hsa-miR-18a-5p, hsa-miR-7-5p, hsa-miR-92b-3p
TNRC6C	hsa-miR-152-3p
TRAM1	hsa-miR-155-5p
UHMK1	hsa-miR-18a-5p, hsa-miR-7-5p
USF2	hsa-miR-92b-3p
XPO1	hsa-miR-205-3p, hsa-miR-455-3p
ZDHHC21	hsa-miR-455-3p, hsa-miR-205-3p
ZDHHC7	hsa-miR-152-3p, hsa-miR-181a-5p
ZNF217	hsa-miR-152-3p

MAPK signaling pathway

gene	miRNA
AGGF1	hsa-miR-6763-5p
AGTRAP	hsa-miR-155-5p
AKAP9	hsa-miR-205-3p
ARAF	hsa-miR-654-5p
ARRB2	hsa-miR-155-5p
BCL2L11	hsa-miR-152-3p, hsa-miR-92b-3p, hsa-miR-181a-5p
BRAP	hsa-miR-181a-5p
CALM1	hsa-miR-181a-5p
CAMK2A	hsa-miR-92b-3p, hsa-miR-654-5p, hsa-miR-3135b
CAMK2D	hsa-miR-7-5p
CAMK2G	hsa-miR-3135b

CLCN6	hsa-miR-152-3p, hsa-miR-3132
CNKSR2	hsa-miR-181a-5p
DUSP10	hsa-miR-92b-3p, hsa-miR-181a-5p
DUSP16	hsa-miR-205-3p, hsa-miR-18a-5p
DUSP9	hsa-miR-1246
ESRP1	hsa-miR-92b-3p
FAM114A2	hsa-miR-7-5p
FAM131B	hsa-miR-7-5p
FXR1	hsa-miR-92b-3p, hsa-miR-155-5p, hsa-miR-7-5p
IQGAP1	hsa-miR-205-3p, hsa-miR-7-5p
ITGB3	hsa-miR-1234-3p
KDM7A	hsa-miR-7-5p, hsa-miR-501-3p, hsa-miR-205-3p, hsa-miR-155-5p, hsa-miR-18a-5p
KIAA1549	hsa-miR-205-3p
KRAS	hsa-miR-96-5p, hsa-miR-501-3p, hsa-miR-7851-3p, hsa-miR-155-5p
KSR1	hsa-miR-7-5p
KSR2	hsa-miR-92b-3p
LMNA	hsa-miR-6763-5p, hsa-miR-96-5p
MAP2K2	hsa-miR-654-5p
MAPK1	hsa-miR-378a-3p
MRAS	hsa-miR-654-5p, hsa-miR-96-5p, hsa-miR-152-3p
NRAS	hsa-miR-3135b, hsa-miR-152-3p
PEBP1	hsa-miR-3174
PPP1CB	hsa-miR-152-3p
PPP1CC	hsa-miR-8071
QKI	hsa-miR-96-5p, hsa-miR-501-3p, hsa-miR-181a-5p
RAF1	hsa-miR-7-5p
RAP1A	hsa-miR-92b-3p
RAP1B	hsa-miR-92b-3p, hsa-miR-181a-5p, hsa-miR-378a-3p
SPRED1	hsa-miR-205-3p, hsa-miR-155-5p
TENT4A	hsa-miR-92b-3p
TLN1	hsa-miR-6763-5p
TRAK1	hsa-miR-181a-5p
YWHAB	hsa-miR-205-3p, hsa-miR-7-5p
ZC3HAV1	hsa-miR-205-3p

TGFb signalling pathway

gene	miRNA
CBL	hsa-miR-7-5p, hsa-miR-7851-3p
CCNC	hsa-miR-3135b, hsa-miR-92b-3p
CCNT2	hsa-miR-7-5p, hsa-miR-155-5p
CGN	hsa-miR-654-5p
COL1A2	hsa-miR-92b-3p, hsa-miR-7-5p
E2F5	hsa-miR-181a-5p, hsa-miR-96-5p
EP300	hsa-miR-96-5p
F11R	hsa-miR-501-3p, hsa-miR-654-5p
FBN1	hsa-miR-152-3p
FKBP1A	hsa-miR-181a-5p
FURIN	hsa-miR-3132
ITGA8	hsa-miR-92b-3p
ITGAV	hsa-miR-92b-3p
ITGB1	hsa-miR-455-3p
ITGB3	hsa-miR-1234-3p
ITGB8	hsa-miR-3132, hsa-miR-181a-5p, hsa-miR-205-3p
LTBP1	hsa-miR-152-3p, hsa-miR-6763-5p
MAPK1	hsa-miR-378a-3p
MEN1	hsa-miR-3135b
NCOR1	hsa-miR-205-3p
NEDD4L	hsa-miR-92b-3p
PARP1	hsa-miR-7-5p
PMEPA1	hsa-miR-92b-3p, hsa-miR-96-5p
PPM1A	hsa-miR-96-5p, hsa-miR-3135b, hsa-miR-654-5p

PPP1CA	hsa-miR-654-5p
PPP1CB	hsa-miR-152-3p
PPP1CC	hsa-miR-8071
PRKCZ	hsa-miR-152-3p
RHOA	hsa-miR-3135b
RNF111	hsa-miR-7-5p
SCG2	hsa-miR-155-5p
SERPINE1	hsa-miR-654-5p
SKI	hsa-miR-654-5p
SKIL	hsa-miR-96-5p, hsa-miR-205-3p
SMAD2	hsa-miR-455-3p, hsa-miR-18a-5p
SMAD7	hsa-miR-96-5p, hsa-miR-92b-3p
SMURF1	hsa-miR-92b-3p
SP1	hsa-miR-205-3p, hsa-miR-7-5p
STUB1	hsa-miR-455-3p
TFDP2	hsa-miR-205-3p, hsa-miR-92b-3p, hsa-miR-155-5p, hsa-miR-501-3p, hsa-miR-96-5p, hsa-miR-152-3p
TGFB1	hsa-miR-18a-5p, hsa-miR-654-5p
TGFB2	hsa-miR-7-5p, hsa-miR-152-3p
TGFBR1	hsa-miR-96-5p
TGIF1	hsa-miR-92b-3p
TGIF2	hsa-miR-181a-5p
TRIM33	hsa-miR-7-5p
UBE2D1	hsa-miR-152-3p
UBE2D3	hsa-miR-7-5p, hsa-miR-181a-5p
USP9X	hsa-miR-654-5p, hsa-miR-155-5p
XPO1	hsa-miR-205-3p, hsa-miR-455-3p
YBX1	hsa-miR-3135b
ZFYVE9	hsa-miR-8071

WNT signaling pathway

gene	miRNA
AGO2	hsa-miR-654-5p
AGO4	hsa-miR-181a-5p, hsa-miR-152-3p, hsa-miR-96-5p, hsa-miR-455-3p
AKT1	hsa-miR-501-3p
AP2A1	hsa-miR-455-3p
AP2B1	hsa-miR-96-5p, hsa-miR-205-3p, hsa-miR-492, hsa-miR-3174
APC	hsa-miR-205-3p
ARRB2	hsa-miR-155-5p
ASH2L	hsa-miR-6763-5p
AXIN2	hsa-miR-205-3p
BCL9	hsa-miR-654-5p, hsa-miR-92b-3p, hsa-miR-455-3p
CALM1	hsa-miR-181a-5p
CAMK2A	hsa-miR-92b-3p, hsa-miR-654-5p, hsa-miR-3135b
CAV1	hsa-miR-205-3p, hsa-miR-96-5p
CCDC88C	hsa-miR-96-5p
CDC73	hsa-miR-1246, hsa-miR-181a-5p, hsa-miR-155-5p
CHD8	hsa-miR-155-5p
CLTB	hsa-miR-6763-5p
CLTC	hsa-miR-96-5p, hsa-miR-455-3p
CSNK1A1	hsa-miR-3174, hsa-miR-6763-5p, hsa-miR-501-3p
CSNK1G2	hsa-miR-155-5p, hsa-miR-378a-3p
CSNK2A1	hsa-miR-3132
CSNK2A2	hsa-miR-654-5p
CTNNBIP1	hsa-miR-92b-3p
CUL3	hsa-miR-92b-3p, hsa-miR-181a-5p, hsa-miR-455-3p
CXXC4	hsa-miR-92b-3p, hsa-miR-8071, hsa-miR-663b
DAAM1	hsa-miR-96-5p, hsa-miR-3174
DACT1	hsa-miR-92b-3p
DKK2	hsa-miR-8071
EP300	hsa-miR-96-5p
FRAT1	hsa-miR-205-3p

FZD3	hsa-miR-96-5p, hsa-miR-205-3p, hsa-miR-18a-5p
FZD4	hsa-miR-7851-3p
FZD5	hsa-miR-155-5p
FZD6	hsa-miR-96-5p
GNAO1	hsa-miR-654-5p
GNB2	hsa-miR-7851-3p
GNB3	hsa-miR-6763-5p
GNB4	hsa-miR-96-5p
GNB5	hsa-miR-152-3p, hsa-miR-455-3p
GNG12	hsa-miR-96-5p
GNG2	hsa-miR-181a-5p, hsa-miR-3132, hsa-miR-18a-5p
GNG3	hsa-miR-654-5p
GNG7	hsa-miR-492, hsa-miR-6763-5p
GSK3B	hsa-miR-18a-5p, hsa-miR-155-5p
ITPR1	hsa-miR-92b-3p, hsa-miR-96-5p
ITPR2	hsa-miR-3132, hsa-miR-96-5p
KMT2D	hsa-miR-1246
KRAS	hsa-miR-96-5p, hsa-miR-501-3p, hsa-miR-7851-3p, hsa-miR-155-5p
KREMEN1	hsa-miR-3135b
KREMEN2	hsa-miR-455-3p, hsa-miR-3135b
LRP6	hsa-miR-96-5p, hsa-miR-455-3p, hsa-miR-7-5p
MEN1	hsa-miR-3135b
NFATC1	hsa-miR-3135b
NLK	hsa-miR-92b-3p, hsa-miR-181a-5p
PDE6B	hsa-miR-7851-3p
PDE6G	hsa-miR-654-5p
PFN1	hsa-miR-6763-5p, hsa-miR-96-5p
PIP5K1B	hsa-miR-205-3p
PLCB1	hsa-miR-205-3p, hsa-miR-378a-3p, hsa-miR-7-5p
PPP2CB	hsa-miR-1246, hsa-miR-155-5p
PPP2R1B	hsa-miR-7-5p
PPP2R5D	hsa-miR-6763-5p
PPP2R5E	hsa-miR-501-3p
PPP3CA	hsa-miR-501-3p
PPP3R1	hsa-miR-181a-5p, hsa-miR-96-5p
PRKACA	hsa-miR-6763-5p
PRKCB	hsa-miR-7-5p
PSMA1	hsa-miR-205-3p
PSMA5	hsa-miR-1246, hsa-miR-378a-3p
PSMB5	hsa-miR-663b
PSMC2	hsa-miR-378a-3p
PSMD10	hsa-miR-205-3p
PSMD11	hsa-miR-3135b, hsa-miR-501-3p
PSMD8	hsa-miR-455-3p
PSMD9	hsa-miR-654-5p, hsa-miR-152-3p
PSME3	hsa-miR-7-5p
PSME4	hsa-miR-492
PYGO2	hsa-miR-96-5p, hsa-miR-3132, hsa-miR-3135b
RAC1	hsa-miR-96-5p, hsa-miR-155-5p
RBBP5	hsa-miR-205-3p, hsa-miR-3135b
RHOA	hsa-miR-3135b
RNF146	hsa-miR-155-5p
RSPO4	hsa-miR-205-3p, hsa-miR-654-5p
RYK	hsa-miR-96-5p, hsa-miR-7-5p
SCG2	hsa-miR-155-5p
SMARCA4	hsa-miR-155-5p
SMURF1	hsa-miR-92b-3p
SNX3	hsa-miR-3135b
SOST	hsa-miR-378a-3p
SOX2	hsa-miR-1246

SOX4	hsa-miR-92b-3p
SOX6	hsa-miR-96-5p, hsa-miR-3135b, hsa-miR-378a-3p, hsa-miR-3132, hsa-miR-181a-5p, hsa-miR-455-3p, hsa-miR-7-5p
SOX9	hsa-miR-155-5p
TCF4	hsa-miR-152-3p, hsa-miR-205-3p, hsa-miR-92b-3p, hsa-miR-7-5p, hsa-miR-155-5p
TCF7L2	hsa-miR-654-5p, hsa-miR-155-5p, hsa-miR-181a-5p, hsa-miR-96-5p
TLE1	hsa-miR-92b-3p
TLE4	hsa-miR-155-5p
TMED5	hsa-miR-92b-3p, hsa-miR-3132, hsa-miR-205-3p
TNKS	hsa-miR-205-3p
TNKS2	hsa-miR-205-3p, hsa-miR-96-5p
TNRC6A	hsa-miR-152-3p, hsa-miR-654-5p, hsa-miR-96-5p, hsa-miR-7-5p
TNRC6B	hsa-miR-18a-5p, hsa-miR-7-5p, hsa-miR-92b-3p
TNRC6C	hsa-miR-152-3p
VPS26A	hsa-miR-96-5p
WIF1	hsa-miR-181a-5p
WLS	hsa-miR-3132
WNT10A	hsa-miR-378a-3p
WNT10B	hsa-miR-152-3p
WNT4	hsa-miR-654-5p, hsa-miR-3135b
WNT5B	hsa-miR-8071
WNT6	hsa-miR-654-5p
XIAP	hsa-miR-96-5p, hsa-miR-205-3p, hsa-miR-455-3p
XPO1	hsa-miR-205-3p, hsa-miR-455-3p
ZNRF3	hsa-miR-155-5p

Supplementary Figure S1

Unsupervised hierarchical clustering heatmap including all expressed miRNAs (n = 1031). p16/HPV DNA status, p16^{INK4A} status and tumour localisation is indicated on a case-by-case basis. Heat map colours indicate mRNA expression z-scores on a scale of -6 to 6.

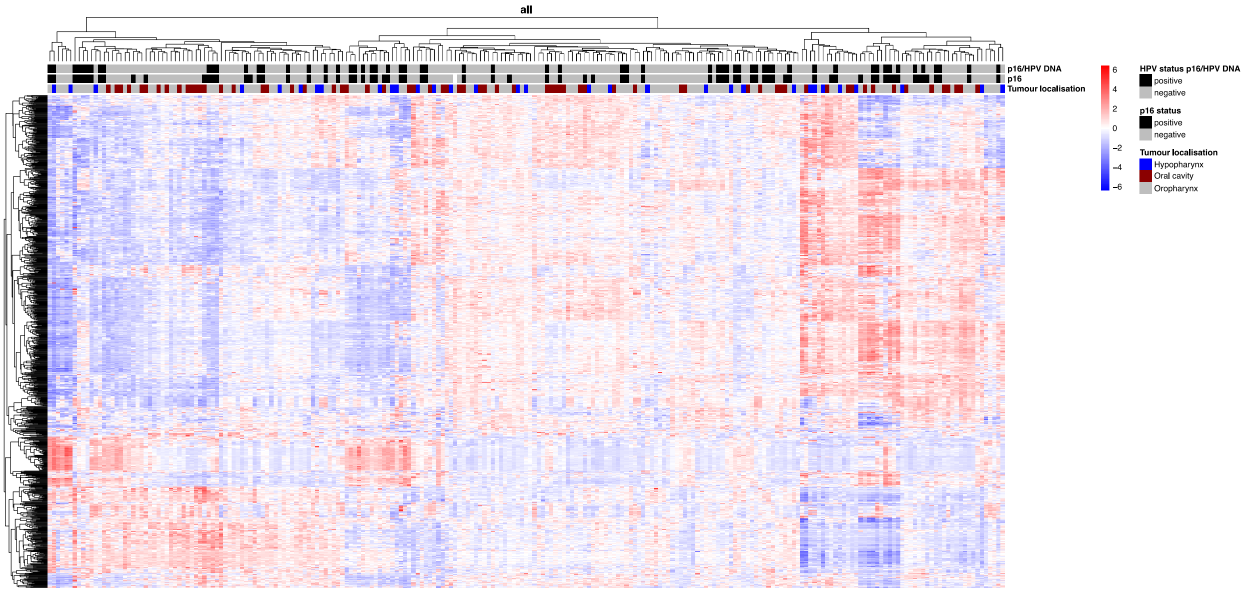

Supplementary Figure S2

Heatmap differentially expressed miRNAs (n = 55; adjusted p-value < 0.05 and |log2 fold change| > 0.5) in HPV-positive (n = 61) versus HPV-negative (n = 168) HNSCC (p16/HPV DNA status). Heat map colours indicate mRNA expression z-scores on a scale of -5 to 5.

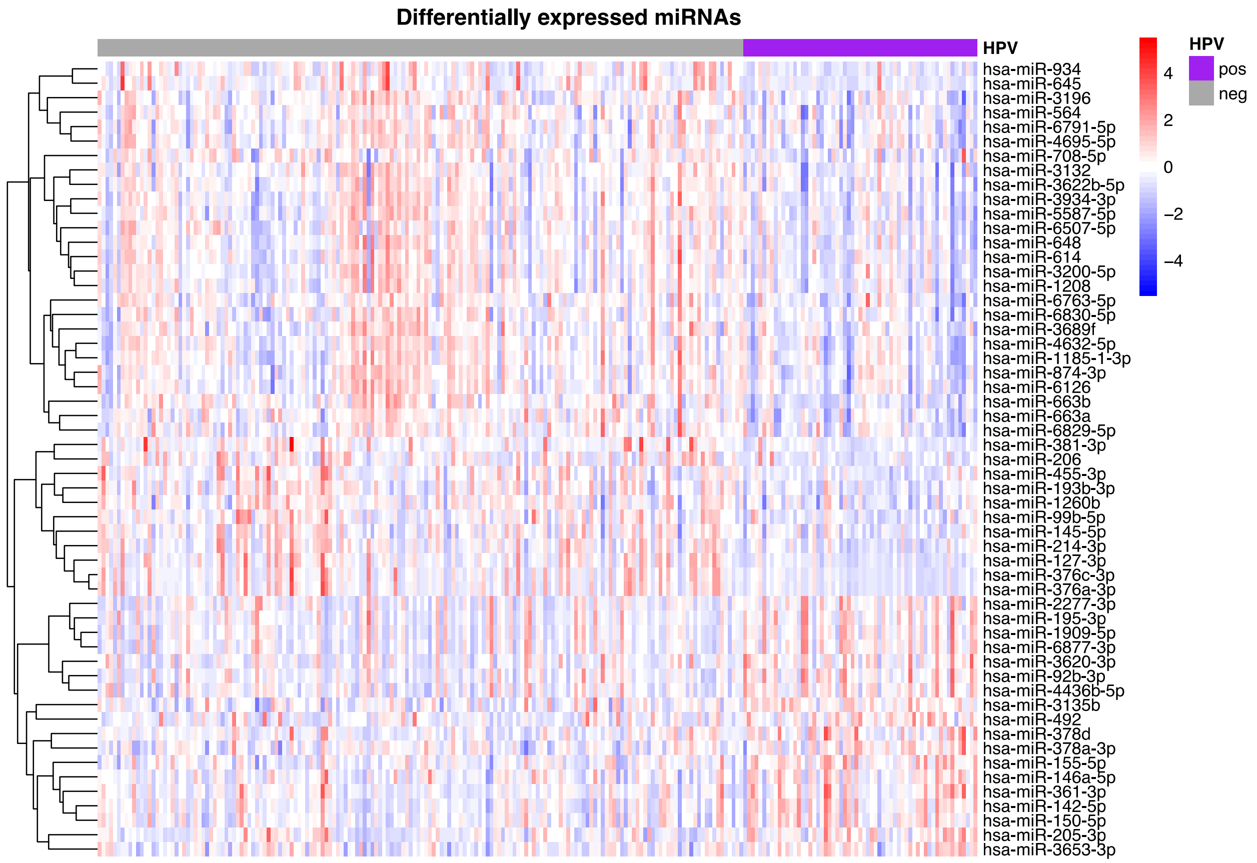

Supplementary Figure S3

Kaplan–Meier curves for the endpoint overall survival according to the 24-miRNA signature predicted HPV-status in **(A)** p16/HPV DNA-positive and **(B)** p16/HPV DNA-negative patients. *P*-values are derived by log-rank test. Hazard ratios (HR) with 95% confidence intervals (CI) are given.

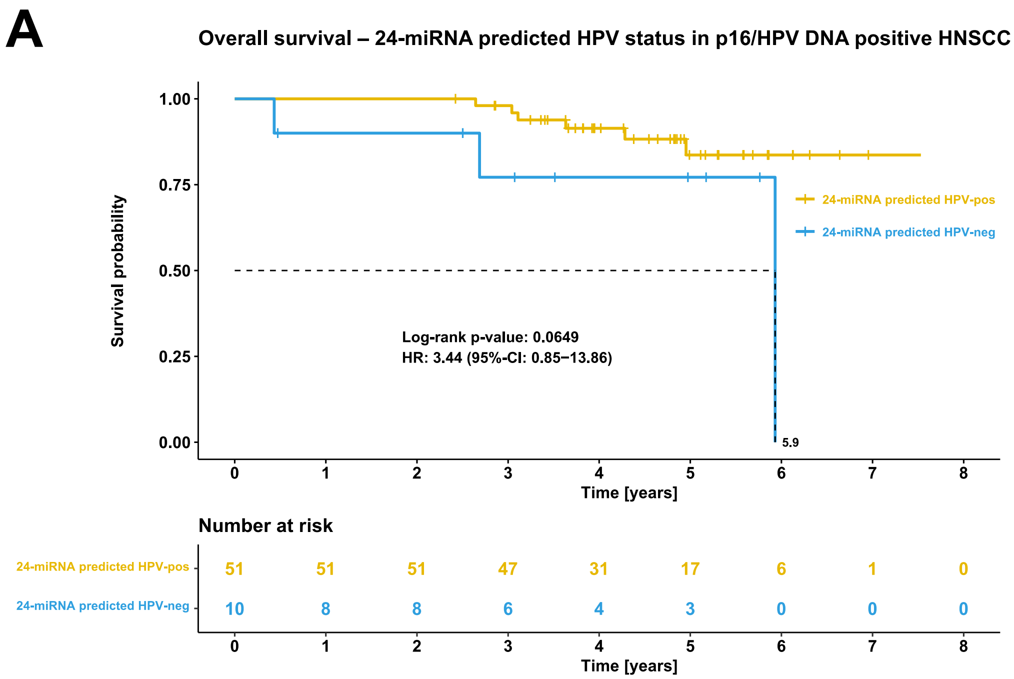

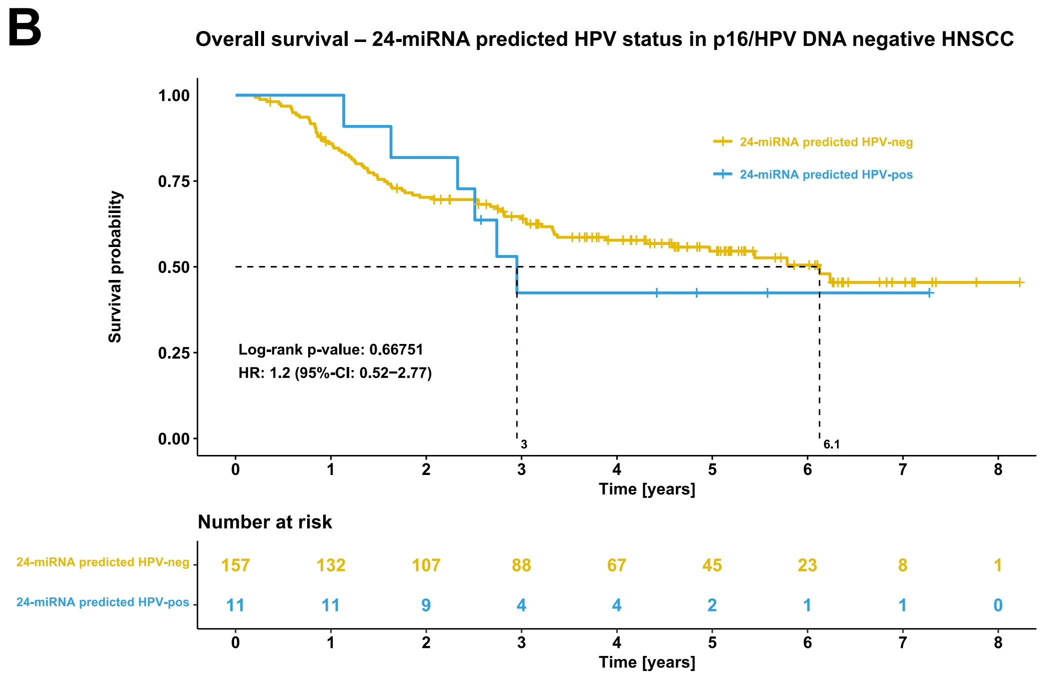

Supplementary Figure S4

Significantly enriched KEGG pathways (adjusted *p*-values < 0.05) of the top 1000 miRNA-mRNA interactions in 24-miRNA-positive/HPV-associated (left) and 24-miRNA-negative/HPV-negative (right) HNSCC: (**A**) Enrichment dot plots of the top 25 KEGG pathways with the highest gene ratios (i.e., the percentage of genes in the given KEGG pathway) and **(B**) enrichment maps. Information on gene count/size (i.e., the number of genes enriched in a KEGG pathway) is given. Overlapping significantly enriched pathways in HPV-positive and HPV-negative HNSCC are written in bold. Significantly enriched KEGG pathways occurring exclusively in 24-miRNA-positive/HPV-associated (left) or 24-miRNA-negative/HPV-negative (right) tumours are underlined (bold blue).

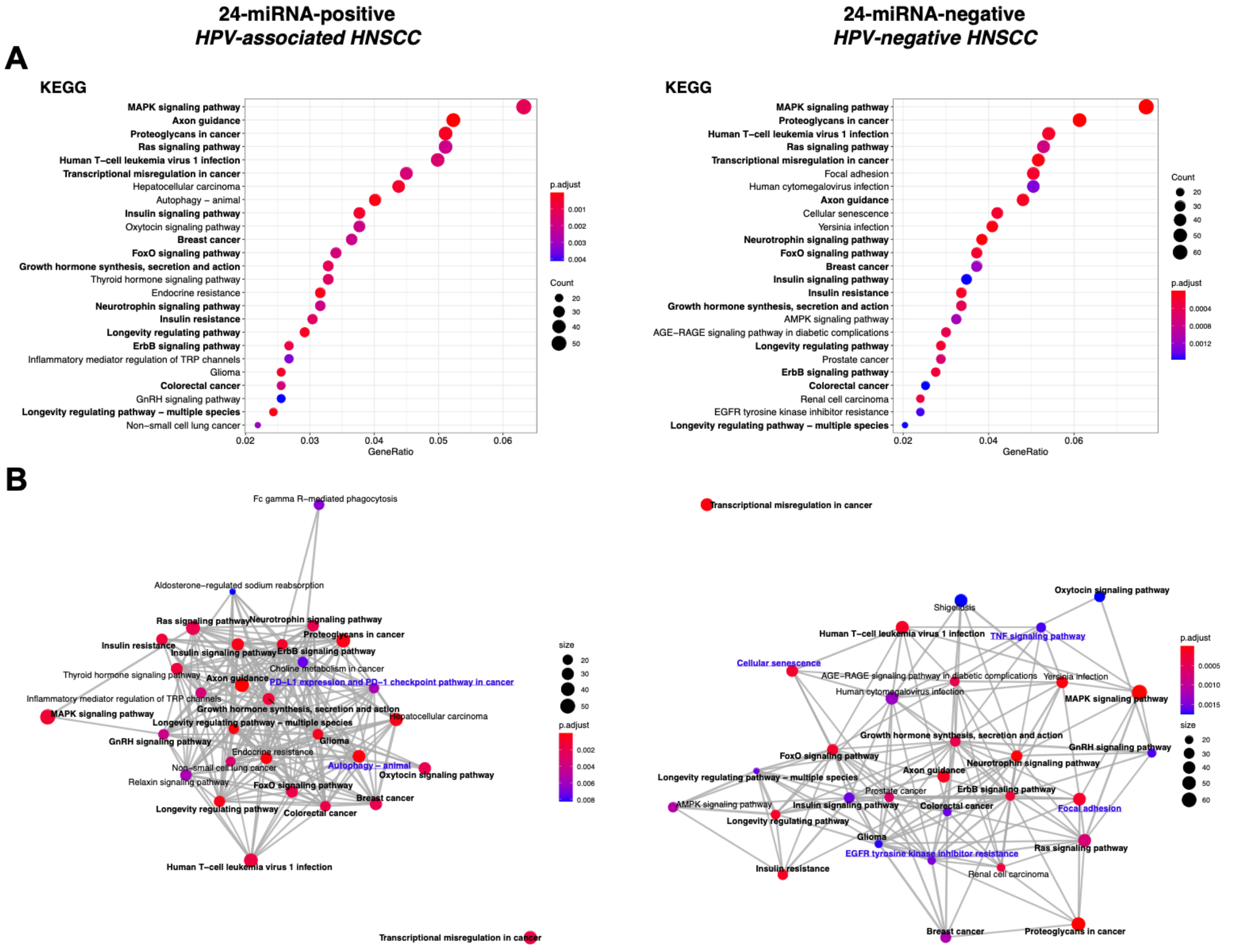

Supplement: Supplementary file 1 [file cancers-14-03745-s001.zip › cancers-1813359-supplementary.pdf]
